# Exploring the molecular mechanism of *Dioscorea alata* L. for the treatment of menstrual disorders using network pharmacology and molecular docking

**DOI:** 10.1016/j.heliyon.2025.e42582

**Published:** 2025-02-08

**Authors:** Rajendran Silambarasan, A. Kasthuri Nair, Gomathi Maniyan, R. Vijaya, Reshma V.R. Nair, J. Hareendran Nair, S. Nishanth Kumar, Shan Sasidharan

**Affiliations:** a Department of R&D, Pankajakasthuri Herbal Research Foundation, Pankajakasthuri Ayurveda Medical College Campus, Trivandrum, India; b Department of Kayachikitsa, Pankajakasthuri Ayurveda Medical College & PG Centre, Killy, Kattakada, Thiruvananthapuram, Kerala, India; c Native Women Food Products Foundation, Research and Development Department, SMIDS Campus, Nagercoil, Tamil Nadu, India; d Department of Dravyagunavijnanam, Pankajakasthuri Ayurveda Medical College & P.G. Centre, Killy, Kattakada, Thiruvananthapuram, Kerala, India; e HCEMM-SU Cardiovascular Comorbidities Research Group, Department of Pharmacology and Pharmacotherapy, Semmelweis University, 1089, Budapest, Hungary

**Keywords:** *Dioscorea alata*, Menstrual disorder, Drug target interaction network, Network pharmacology, Protein-protein interaction and *in-silico* analysis

## Abstract

Menstrual disorders (MDs), including premenstrual syndrome, amenorrhea, and dysmenorrhea, affect women globally. *Dioscorea alata* L., a traditional yam species, has been used medicinally, but its potential in treating MDs remains understudied. This study employs a network pharmacology approach to examine the effects of *D. alata's* secondary metabolites on MDs via multi-target mechanisms. Compounds were identified from literature and PubChem, while disease-related targets were gathered from GeneCards, DisGeNET, and CTD databases. Swiss target prediction was used to link compounds to targets. A protein-protein interaction (PPI) network was constructed using STRING, and Gene Ontology (GO) and KEGG enrichment analyses were conducted to predict functional pathways. Eighteen bioactive compounds and 120 therapeutic targets specific to MDs were identified. KEGG analysis revealed 20 significant pathways related to menstrual disturbances. Among the 120 targets, TNF α, PPARG, ESR1, and AKT1 were highlighted as key therapeutic targets. Molecular docking showed strong interactions between Daidzein and ESR1, Diosgenin and TNF α, Alatanin and AKT1, and PPARG. The findings suggest that *D. alata's* bioactive compounds, such as Diosgenin, Daidzein, Genistin, Cycloartane, and Alatanin, could modulate pathways involved in ovarian follicle formation, hormone regulation, estrogen receptor signaling, and the stress-activated MAP kinase pathway. This study provides new insights into the multi-target potential of *D. alata* for treating menstrual disorders, supporting further investigation and therapeutic development.

## 1 Introduction

Menstrual disorders (MDs) and reproductive health concerns are common physiological issues affecting women worldwide, with the majority of women of reproductive age experiencing them [1]. Common MDs include premenstrual syndrome (PMS), dysmenorrhea, amenorrhea, menorrhagia, oligomenorrhea, irregular periods, and leucorrhea (white discharge) [2,3]. Approximately 75 % of women worldwide are affected by menstrual problems. According to a systematic analysis by the World Health Organization, the incidence of dysmenorrhea among women of reproductive age ranges from 16.8 % to 81.0 %, with severe symptoms observed in 12–14 % of cases [4]. Out of common MDs, PMS affects 30–40 % of the fertile female population [5]. Among reproductive-aged women, polycystic ovarian syndrome (PCOS) is a common endocrine condition characterized by polycystic ovarian dysfunction, increased androgen levels, and ovulatory dysfunction. Infertility and irregular menstruation cycles are primarily caused by it. World Health Organization estimates that between 8 and 13 % of women in this age group have polycystic ovary syndrome (PCOS), with as many as 70 % of those women dealing with menstrual cycle irregularities and other reproductive health issues [6]. New research suggests that endometrial lesions are typically present in women with polycystic ovary syndrome (PCOS) who also have subfertility or chronic pelvic pain [7].

Additionally, itching and foul odor are common issues experienced during and after the menstrual cycle [8]. Studies have highlighted significant variations in symptoms among women with MDs residing in different regions, suggesting that factors such as dietary habits, lifestyle choices, cultural norms, and personal constitutions play a crucial role in the manifestation of these conditions [9]. In contemporary medical practice, hormone therapy is the primary treatment for MDs. In addition, the management of menstruation diseases encompasses various treatments, including nonsteroidal anti-inflammatory drugs, lifestyle modifications, and surgical interventions, tailored to the unique condition and patient requirements. However, due to the adverse effects associated with modern medicine, an increasing number of women are opting for herbal remedies rooted in traditional or national medicinal practices [10].

The larger yam (*Dioscorea alata* L.), also known as water yam or greater yam, is the most frequently cultivated yam species worldwide [11]. *D. alata* tubers play a vital role in food security, medicine, and the economies of developing nations, ranking as the fourth most important and widely used root and tuber crop globally. The tubers of *D. alata* contain several bioactive biomolecules, including diosgenin, dioscorine, dioscin, allantoin, crude fat, crude fiber, catechins, chlorogenic acids, and proanthocyanidins [[11], [12], [13], [14], [15]]. The pharmacological effects of *D. alata* include anti-diabetic, anti-hypertensive, antioxidant, anti-apoptotic, anti-infective, anti-cancer, antimicrobial, cardioprotective, hypolipidemic, and hypocholesterolemia activities [16]. One of the key areas where *D. alata* shows promise is in menstrual health, primarily due to its bioactive compounds [17]. In traditional medicine systems, *D. alata* is recognized for its diverse bioactive compounds, which play a role in alleviating menstrual symptoms such as dysmenorrhea, irregular cycles, and other related conditions [18]. One of the notable bioactive compounds found in *D. alata* is diosgenin, which has estrogenic properties [19,20]. This compound is believed to regulate hormonal imbalances, contributing to more regular menstrual cycles and reducing symptoms of PMS and dysmenorrhea [21]. Additionally, compounds like alatanins and daidzein present in *D. alata* possess antioxidant and anti-inflammatory properties, which are crucial in managing inflammatory conditions linked to menstrual pain and disorders [22]. Furthermore, *D. alata* compounds have implications for menopausal health. Phytoestrogens found in the *D. alata*, such as genistein, mimic estrogen's effects in the body, potentially reducing menopausal symptoms such as hot flashes and mood swings [23]. These compounds may also contribute to maintaining bone health during menopause, offering a natural alternative to conventional hormone replacement therapies [24,25]. Due to these properties, *D. alata* emerges as a natural source of bioactive compounds with significant potential to enhance women's health across different stages of life. From alleviating MDs and menopausal symptoms to supporting metabolic and hormonal balance, ongoing research underscores its role as a valuable resource in promoting women's well-being. In traditional practices, *D. alata* is often prepared and consumed in various forms, such as decoctions, teas, or as part of dietary supplements [15,26]. These preparations are aimed at harnessing the plant's medicinal properties to support women's reproductive health. One of the key challenges in understanding the effects of *D. alata* on menstrual health is the lack of focused studies examining its influence on hormonal balance. Hormonal regulation is crucial for managing menstrual health, and while *D. alata* contains bioactive compounds that may affect estrogenic activity, its specific role in modulating menstrual cycles remains unclear. This gap in knowledge often leads to an overgeneralization of its broader anti-inflammatory or metabolic effects as directly beneficial for menstrual health, despite limited targeted evidence. However, diosgenin [27] and daidzein [28] interact with inflammation and hormone-regulating mechanisms provides a more comprehensive understanding of how *D. alata* could be beneficial in managing menstrual-related conditions. This approach clarifies the pharmacological role of these compounds and strengthens the scientific basis for their use in reproductive health treatments. In addition, there is a lack of appropriate *in vitro* and *in vivo* research on *D. alata* for treating MDs in women. Further exploration through scientific studies is crucial to fully harnessing the therapeutic benefits of *D. alata* for women's health.

Network pharmacology is an interdisciplinary field that combines systems biology and bioinformatics to explore the molecular interactions between drugs and therapeutic targets comprehensively [29]. This approach provides a systems-level and holistic perspective, allowing for a more comprehensive understanding of the intricate mechanisms underlying drug action [30]. Network pharmacology advances drug development and clinical interventions by clarifying the systemic effects of existing drugs. In the era of big data and artificial intelligence, it represents an innovative field in systematic drug research [31,32]. Using computational techniques, network pharmacology examines pharmaceutical benefits, side effects, and predicts mechanisms of action. It is increasingly applied in traditional and ethnic medicine to identify key chemical components and their target functions by constructing "drug ingredient-target" networks, linking chemical components in TCM formulas to disease targets [33]. Network pharmacology has emerged as a transformative approach in understanding the multifaceted interactions within Ayurvedic medicine, facilitating the integration of traditional knowledge with modern biomedical science. By employing systems biology and bioinformatics, network pharmacology deciphers the complex relationships between bioactive compounds, molecular targets, and therapeutic effects inherent in Ayurvedic formulations [34]. In addition, network pharmacology studies underscore the potential of network pharmacology in bridging traditional Ayurvedic medicine with contemporary scientific research, offering a comprehensive understanding of the molecular mechanisms underlying Ayurvedic formulations and paving the way for the development of novel therapeutic strategies [35]. This approach reveals how traditional formulas treat complex diseases and aids in screening active ingredients in traditional medicine, validated through *in vitro* and *in vivo* experiments [36]. It also helps identify new active compounds, integrating modern pharmacology with traditional medicine theory to enhance the understanding of traditional practices and expand their scientific framework [37].

This study aims to fill this gap by using network pharmacology, bioinformatics, and *in silico* molecular docking experiments to predict and investigate the critical targets, bioactive components, and mechanisms of *D. alata* bioactive molecules in combating menstrual problems in women. This research provides a foundation for further in-depth investigations of the bioactive compounds and their potential therapeutic applications. The key targets and constituents of *D. alata* were subsequently validated.

## 2 Materials and methods

### 2.1 Screening of components

The chemical constituents of *D. alata* were obtained from PubChem, a comprehensive database of chemical compounds and their biological test responses (Supp. Table 1) [38] (https://pubchem.ncbi.nlm.nih.gov/). PubChem also provides detailed structural data for these compounds. To filter the ingredients, oral bioavailability (OB) and drug-likeness (DL) were used as criteria. Oral bioavailability is a critical pharmacokinetic metric for orally administered medications, indicating the extent to which a molecule is absorbed into the bloodstream [39].

### 2.2 Prediction of pharmacological and ADME/T properties

The ADME/T properties of compounds were assessed using the Swiss Target prediction and pkCSM (https://biosig.lab.uq.edu.au/pkcsm/) [40]. The absorption phase includes metrics such as polar surface area (PSA), membrane permeability (Log P), and various cell-based assays like Caco-2 and intestinal absorption. Distribution metrics include blood-brain barrier permeability (log BB) and central nervous system (CNS) permeability. Drug metabolism is studied using cytochrome models to predict substrate interactions. Excretion focuses on the elimination of ineffective drugs/metabolites from the body. Toxicity is evaluated through parameters like AMES toxicity, hERG inhibition, hepatotoxicity, and skin sensitization. Ideal compounds are expected to have good intestinal absorption and log Kp values. The ability to cross the blood-brain barrier (BBB) is considered, where log BB > 0.3 indicates easy passage, and log BB < -1 suggests no passage. CNS penetration is similarly assessed, with log PB > -2 suggesting penetration and <-3 indicating no penetration. Key descriptors such as molecular weight, dipole moment, SASA, hydrogen bond donor/acceptor properties, Lipinski's rule, and human oral absorption are also predicted and considered for selecting optimal compounds (Supp. Table 2) [41].

### 2.3 The prediction of putative targets of the selected compounds

The targets related to the selected chemical components of *D. alata* were predicted using several databases, including TCMSP, BindingDB [42] (https://www.bindingdb.org/bind/index.jsp), ChEMBL [43](https://www.ebi.ac.uk/chembl/), and Swiss Target Prediction [44] (http://www.swisstargetprediction.ch/), with the species limited to *Homo sapiens*. These predictions were based on the structure similarities between small drug-like compounds and their targets. The UniProt protein sequence library [45] (http://www.uniprot.org/) was used to verify the collected target information. Only proteins that directly interacted with each chemical component in *D. alata* were selected as potential targets.

### 2.4 The prediction of known *D. alata* therapeutic targets acting on MDs

The targets related to MDs were obtained from two publicly accessible databases. The first database used was DrugBank, a unique bioinformatics and cheminformatics resource that integrates drug and target data in an encyclopedic format [46]. Only interactions between drugs and human gene/protein targets approved by the US Food and Drug Administration (FDA) for the treatment of MDs were selected. The second database was DisGeNET, a comprehensive and reliable knowledge base for human variant-disease associations and gene-disease associations, used as a data collection tool for genes linked to diseases.

We searched these databases using the following queries: "irregular menstruation," "heavy menstrual bleeding," "hypomenorrhea," "amenorrhea," "dysmenorrhea," "oligomenorrhea," "ovarian failure premature," "ovarian cysts," "polycystic ovary syndrome," and "menopausal syndrome." The targets related to MDs were then filtered out to focus on the clinical applications of *D. alata* in the treatment of MDs.

### 2.5 Bioactive compound-target-disease network construction

The "bioactive compound-target-disease" network was constructed using common target data and subsequently uploaded to Cytoscape v3.10.2 (Boston, MA, USA).

### 2.6 Protein-protein interaction (PPI) network construction

To investigate the multiscale molecular processes of herbal components used in the treatment of MDs, a PPI network was constructed using data from the STRING database [47,48]. The organism selected was ∗*Homo sapiens*∗, with a minimum required interaction score set at 0.7. To identify closely linked network modules, the Molecular Complex Detection (MCODE, version 2.0.2) plugin in Cytoscape was utilized. Additionally, the maximum clique centrality (MCC) method, implemented via the Cytoscape plugin cytoHubba (version 0.1), was used to identify highly linked core targets, providing a more accurate identification of important proteins within the PPI network.

### 2.7 Enrichment analyzing core targets by GO and KEGG

For gene annotation, enrichment, and pathway analysis, the STRING was utilized. To analyze the core targets, Gene Ontology (GO) enrichment analysis was performed. The top 10 GO terms with the lowest P-value were selected for each of the three GO categories: Molecular Function (MF), Cellular Component (CC), and Biological Process (BP). Additionally, the list of genes of interest was uploaded into STRING for KEGG pathway enrichment analysis. The top 20 enriched pathways with the lowest P-value, or the highest log10 (P value), were selected for the development of the Compound-Target-Pathway network [47].

### 2.8 Inferring upstream pathway activity

The understanding of aberrant transcriptome regulation relies significantly on the activity of signal transduction pathways derived from transcriptomic data. Therefore, to assess the activity of upstream pathways shared by MDs and *D. alata*, we employed SPEED2 [49]. This platform provides insights into sixteen signaling pathways potentially implicated in the dysregulation of these shared genes [50].

### 2.9 Molecular docking

To validate the accuracy of the predicted hub genes, molecular docking was performed using Pyrx Autodock Vina [51,52]. The three-dimensional structures of these targets were retrieved from the PDB database [53,54], while the chemical structures of all bioactive compounds were obtained from PubChem [38,55]. Docking scores were utilized to assess the potential binding capabilities between the bioactive compounds and the hub genes.

## 3 Results

From *Dioscorea alata*, a total of 18 compounds were identified using data sourced from TCMSP, PubMed, and other published references. Among these, 10 compounds met the screening criteria, having oral bioavailability (OB) greater than 30 %, compliance with Lipinski's rule of five (0 or 3 violations), and a drug-likeness (DL) score of ≥0.18. Compounds such as cinnamyl cinnamate, bumetrizole, p-coumaric acid, kaempferol, 8-epidiosbulbin E acetate, catechin, epicatechin, genistein, 1-feruloylglycerol and daidzein acetate were selected based on their adherence to Lipinski's rule with 0 violations. In contrast, six compounds—genistin, alpha-tocopherol, gamma-tocopherol, diosgenin, cycloartane, and 9,12-octadecadienoic acid—exhibited a violation level of 1, while two remaining molecules, alatanin C and delphinidin 3-glucoside chloride, had a violation level of 3.

### 3.1 Therapeutic target prediction

A total of eighteen compounds, along with 120 known therapeutic targets and 300 disease characteristics related to MDs, were identified through data retrieved from the DrugBank and OMIM databases, supplemented by predictions from Swiss Target Prediction.

**Fig. 1:**
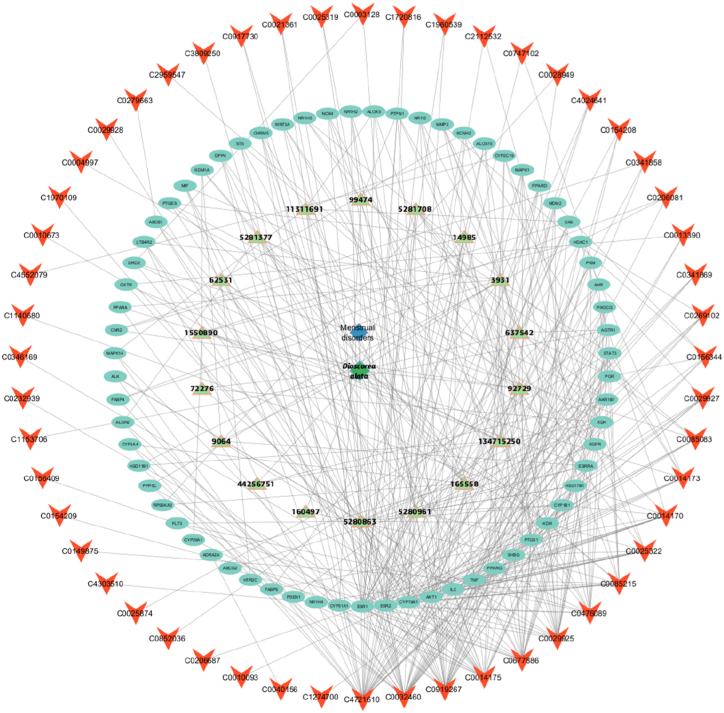
*D. alata* active ingredient-target diagram. Here the green colour indicated the phytomolecules of *D. alata*, sky blue color indicates the genes and red colour arrow indicated the diseases category.

A bioactive compound-target-disease network was constructed using Cytoscape software, encompassing 139 nodes and 437 edges. The network topology was analyzed using the Network Analyzer module in Cytoscape, which facilitated the determination of the R value for each node, with higher R values indicating more significant nodes within the network. Among the studied compounds, kaempferol, Genistein, 8-epidiosbulbin E acetate, gamma-Tocopherol, p-coumaric acid and 9,12-Octadecadienoic acid were connected with genes involved in MDs and these compounds were found with maximum degree value. The analysis identified the potential active ingredients of *D. alata* for the treatment of MDs as follows: 9,12-Octadecadienoic acid, Alatanin, Alpha-Tocopherol, Bumetrizole, Cianidanol, Cinnamyl Cinnamate, Cycloartane, Daidzein, Delphinidin 3-glucoside, Diosgenin, Epicatechin, Epidiosbulbin E acetate, Gamma-Tocopherol, Genistein, Genistin, 1-feruloyl glycerol, Kaempferol, and p-Coumaric acid (Fig. 1; Supp. Table 3).

The significant therapeutic targets of *D. alata* in MD therapy were identified as TNF, PPARG, AKT1, ESR1, MPK14, MDM2, PTPN1, PGR, ESR2, MPK1, EGFR, IL2, STAT3, KDR, MMP2, HDAC1, CYP1B1, OXTR, CYP26A1, SHBG, and ESRRA **(. 2A)**. These targets are crucial for the biological activity of *D. alata* in addressing MDs, highlighting its potential mechanisms of action through these key molecular interactions.

### 3.2 PPI network

**Fig. 2:**
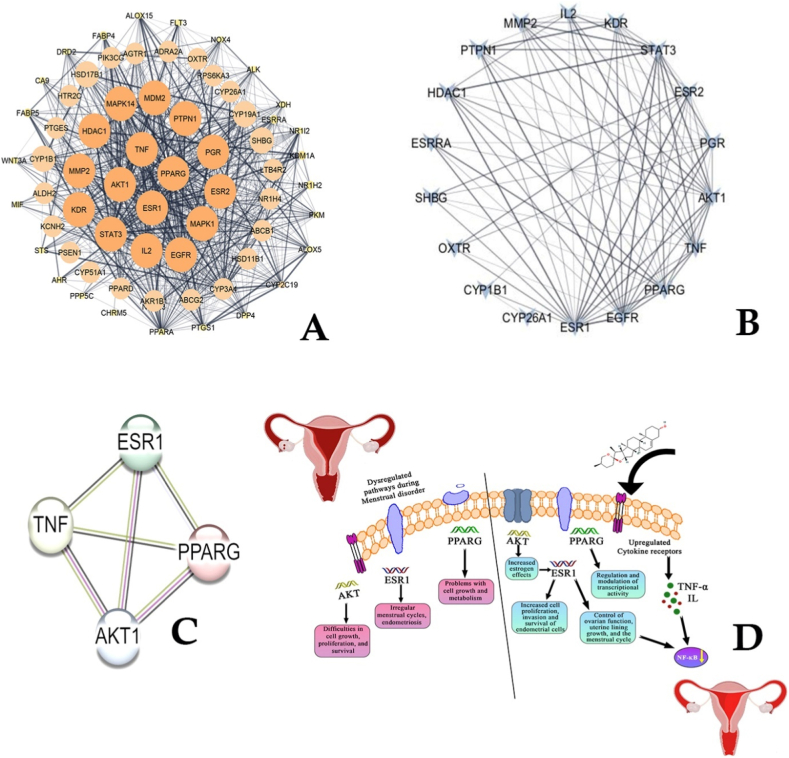
Core target of *D. alata* against MDs. **A** Protein-protein interaction (PPI) network imported from the STRING database into Cytoscape 3.8.0. B. Refined PPI network highlighting more significant proteins. C. Top 4 hub genes identified in the PPI network. F. Diagramatic representation showed, that the molecules regulating hub genes in menstrual disorders.

To elucidate the mechanisms underlying *D. alata* efficacy against MDs (MDs), a PPI network was constructed using data from the STRING database (Fig. 2B). Employing a previously established filtering approach [23], the four hub genes with the highest degree values were identified: AKT (serine/threonine kinase 1), estrogen receptor 1 (ESR1), tumor necrosis factor (TNF), and peroxisome proliferator-activated receptor gamma (PPARG) (Fig. 2C; Supp. Table 3.1). Detailed functional analysis revealed that these hub genes predominantly function as kinases, enzymes, nuclear receptors, nucleic acid binding proteins, and transcription factors. Moreover, diagrammatic representation of Molecules and genes were noticed in Fig. 2D. Here the selected genes are upregulated by the incorporation of D. alata molecules. Furthermore, an interaction network centered around these hub genes was constructed, highlighting their pivotal roles in mediating the biological activities of *D. alata* in treating MDs. This network provides insights into the molecular interactions and pathways through which *D. alata* exerts its therapeutic effects, offering a framework for further mechanistic investigations.

### 3.3 GO enrichment and KEGG pathway analysis

**Fig. 3:**
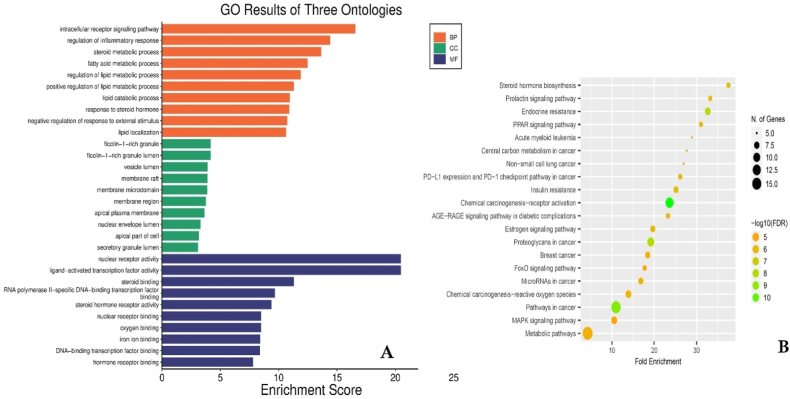
3A. Gene Ontology (GO) analysis for biological processes, cellular components, and molecular functions performed on these genes, with the top ten terms (P < 0.05) displayed. 3B. KEGG enrichment analysis of common targets for MDs treated with *D. alata*.

**Fig. 4:**
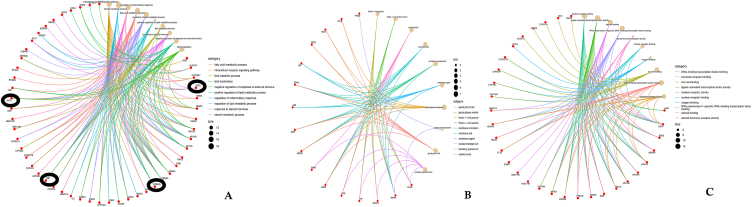
4A. showed the genes involved in the pathway. 4B. showed genes involved in the cellular component and 4C showed the genes involved in the molecular functions.

GO and KEGG pathway enrichment analyses were conducted using data from the STRING database, prioritizing pathways based on false discovery rate (FDR) values below the threshold of <1.0e-16 (Fig. 3, Fig. 4). A total of 3601 GO terms were identified, comprising 3075 Biological Process (BP), 334 Molecular Function (MF), and 192 Cellular Component (CC) terms (Fig. 3A, Fig. 4A, Fig.4B and Fig. 4C & Supp. Table 4.1, 4.2 & 4.3). The GO categorical results highlighted significant involvement of the identified targets in various biological processes. Key processes included intracellular receptor signalling pathway (P = 3.96821E-15; linked with 17 target genes), regulation of inflammatory response (P = 3.97E-15; linked with 18 genes), steroid metabolic process (P = 2.27782E-14; linked with 16 genes), fatty acid metabolic process (P = 3.45203E-13; linked with 16 genes), regulation of lipid metabolic process (P = 1.25582E-12; linked with 16 genes), response to steroid hormone (P = 1.22661E-11; linked with 14 genes), negative regulation of response to external stimulus (P = 1.87027E-11; linked with 16 genes), etc.

In terms of molecular functions, nuclear receptor activity (P = 5.64528E-19; linked with 13 genes), ligand-activated transcription factor activity (P = 5.64528E-19; linked with 13 genes), steroid binding (P = 5.64528E-10; linked with 10 genes),RNA polymerase II-specific DNA-binding transcription factor binding (P = 1.72934E-08; linked with 12 genes), steroid hormone receptor activity (P = 2.73433E-08; linked with 6 genes), nuclear receptor binding (P = 1.87027E-11; linked with 16 genes) etc., Moreover, the proteins associated with these targets were distributed across diverse cellular components, ficolin-1-rich granule (P = 6.66121E-05; linked with 5 genes), ficolin-1-rich granule lumen (P = 6.66121E-05; linked with 5 genes), vesicle lumen (P = 0.000128436; linked with 7 genes), membrane raft (P = 0.000130876; linked with 7 genes), membrane microdomain (P = 0.000133354; linked with 7 genes).

To further verify that the biological processes related to the target proteins are associated with the occurrence of MDs, a total of 10 pathways (p ≤ 0.05) were screened using KEGG pathway analysis. Chemical carcinogenesis - receptor activation (P = 2.8024E-08; linked with 12 genes), Proteoglycans in cancer (P = 1.61597E-07; linked with 11 genes), Endocrine resistance (P = 4.31464E-07; linked with 8 genes), AGE-RAGE signaling pathway in diabetic complications (P = 5.03691E-07; linked with 8 genes), Insulin resistance (P = 9.05653E-07; linked with 8 genes), Steroid hormone biosynthesis (P = 4.6103E-06; linked with 6 genes), Prolactin signaling pathway (P = 1.02188E-05; linked with 6 genes) Fig. 3B & Supp Table 4.4. The results of GO and KEGG analyses suggested that analyses provide valuable insights into the molecular mechanisms through which *D. alata* compounds may exert therapeutic effects against MDs, offering a foundation for further experimental validation and therapeutic development. (see Fig. 4)

### 3.4 Activity in the upstream pathway

**Fig. 5:**
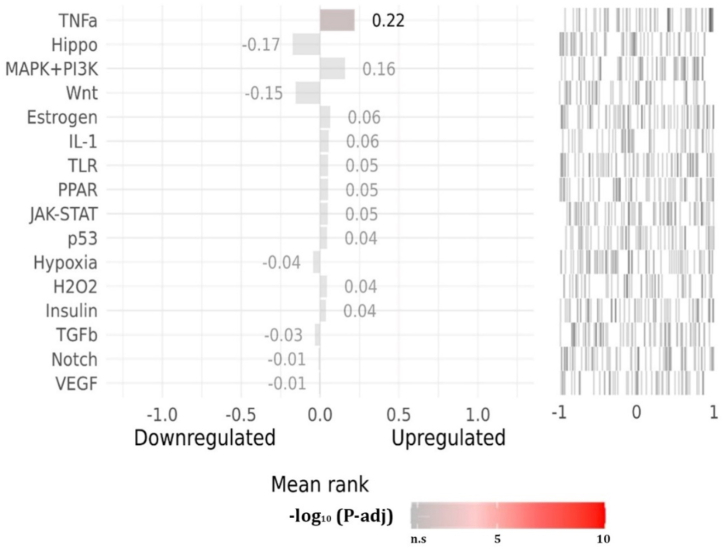
Pathway activity ranking (adjusted P-value <0.05). The colors indicate adjusted P-value, with brighter colors representing higher rankings.

SPEED2 analysis provided a robust approach to assess signaling pathway activity among groups of dysregulated genes identified through transcriptome data analysis. This method facilitated the exploration of upstream regulatory mechanisms associated with similar targets. The heatmap colors represented adjusted P-values, with brighter colors indicating higher-ranked activities, highlighting estrogen's prominent influence in the regulatory networks. Following pathway perturbations, we examined the consistent up-regulation and down-regulation of genes. Genes with adjusted P-values greater than zero were predominantly up-regulated, indicating heightened activity in pathways such as TNF-α, MAPK/ERK, PI3K/Akt, estrogen, IL1, and TLR signaling pathways. Conversely, pathways such as TGF-β, Hippo, and Wnt showed down-regulation (Fig. 5; Table 1). These findings underscore the pivotal roles of estrogen-related signaling pathways and immune response pathways in the regulatory mechanisms influenced by *D. alata* bioactive compounds. The comprehensive analysis enhances our understanding of the molecular dynamics involved in potential therapeutic interventions for MDs, warranting further experimental validation and clinical exploration.

**Table 1:** Pathway activity ranking for the selected targets.

Pathway	Gene	Regulation	Nexp	Zrank	Pval	Qval
Estrogen	PTGES	UP	38	0.42	1.6E-06	0.00043
Estrogen	PGR	UP	17	0.61	1.9E-06	0.00048
Estrogen	FABP5	UP	28	0.47	3.8E-06	0.00092
Hippo	AHR	DOWN	17	−0.56	0.000016	0.0049
Hippo	HDAC1	DOWN	18	−0.49	0.00011	0.015
Hypoxia	MIF	UP	35	0.4	0.000018	0.0031
IL-1	TNF	UP	2	1	0.000002	0.00041
Insulin	STAT3	UP	33	0.47	6.10E-07	0.00042
Insulin	PPARD	UP	34	0.4	0.000018	0.004
Insulin	CYP51A1	UP	12	0.64	0.000022	0.0046
JAK-STAT	STAT3	UP	104	0.35	1.10E-10	0.000016
p53	ALOX5	UP	3	0.99	5.60E-07	0.001
p53	CYP1B1	UP	8	0.67	0.00022	0.032
PPAR	FABP4	UP	12	0.73	7.80E-07	0.0002
PPAR	TNF	DOWN	10	−0.77	1.3E-06	0.00032
PPAR	ABCG2	UP	16	0.62	2.3E-06	0.00053
PPAR	FABP5	UP	19	0.53	0.000017	0.0023
TGFb	CYP19A1	DOWN	16	−0.51	0.00012	0.013
TLR	TNF	UP	101	0.85	5.20E-71	4.20E-07
TLR	PTPN1	UP	104	0.63	5.70E-34	4.20E-07
TNF α	TNF	UP	15	0.76	4.30E-09	0.000011
TNF α	PSEN1	UP	44	0.46	1.70E-08	0.000014
TNF α	HSD11B1	UP	24	0.6	3.90E-08	0.000018
Wnt	AHR	DOWN	13	−0.58	0.000064	0.04

### 3.5 Molecular docking validation

**Fig. 6:**
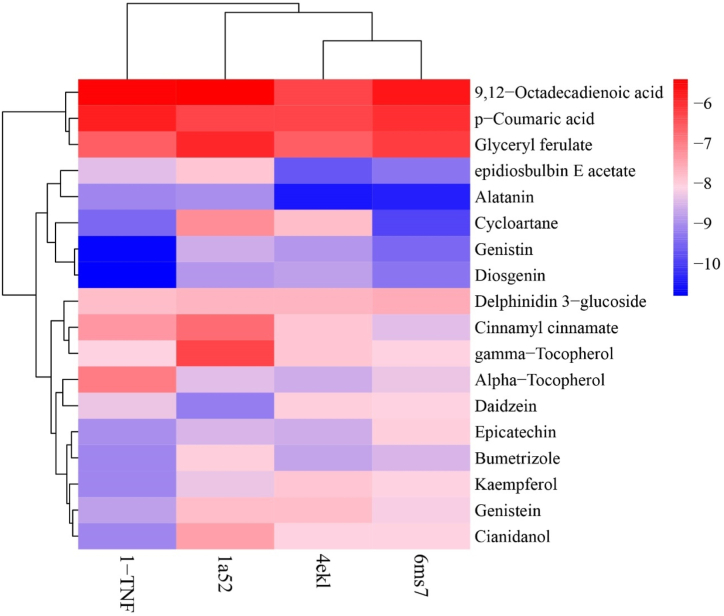
Heat map showing the differences in binding energy between potential bioactive components and the top 4 core targets. Dark blue indicates compounds with higher binding affinity, while pink indicates molecules with lower affinity.

Molecular docking simulations were employed to evaluate the binding capabilities of bioactive compounds from *D. alata* to the identified hub genes associated with MDs. The analysis, depicted in the Two-way cluster analysis heatmap (Fig. 6), demonstrated strong binding affinities between a majority of *D. alata* chemicals and the hub genes. This underscored the pivotal role of these hub genes—AKT1, TNF, ESR1, and PPARG—in mediating the therapeutic effects of *D. alata* against MDs. As illustrated in Fig. 6 and detailed in Supp. Table 5, specific compounds exhibited notable binding affinities: Daidzein showed high binding capacity with ESR1 (score = 9.2), while Diosgenin exhibited significant binding ability with TNF (score = −10.8). Alatanin demonstrated robust binding affinities for both AKT1 (score = −10.5) and PPARG (score = −10.4). The Two-way cluster analysis further categorized molecules such as Alatanin, Cycloartane, Diosgenin, Daidzein, Genistin, and Epidiosbulbin E acetate into distinct clusters based on their binding affinities. Conversely, molecules with lower binding affinities formed another cluster at the top of the heatmap. Additionally, the column annotation cluster highlighted close interactions of proteins such as 6ms7, 4ekl, and 1-TNF with molecules exhibiting maximum binding affinity, indicating robust inhibitory effects of *D. alata* compounds on MDs-associated genes. These findings provide mechanistic insights into the potential therapeutic efficacy of *D. alata* bioactive compounds in treating MDs, underscoring their promising role as inhibitors targeting key molecular pathways. Further experimental validation and clinical studies are warranted to substantiate these computational findings and advance their translational application in clinical settings.

The selection of the optimal docked ligand molecule was based on meticulous evaluation of binding energy, hydrogen bonding characteristics, and alignment with specific amino acid residues. This study focused on assessing interactions between a diverse array of molecules found in *D. alata* and four pivotal targets implicated in MDs in women. Our findings, detailed in Fig. 6 and Supp. Table 5, highlight the significant binding interactions of compounds such as 9,12-Octadecadienoic acid, Alatanin, Alpha-Tocopherol, Bumetrizole, Cianidanol, Cinnamyl cinnamate, Cycloartane, Daidzein, Delphinidin 3-glucoside, Diosgenin, Epicatechin, epidiosbulbin E acetate, gamma-Tocopherol, Genistein, Genistin, 1-feruloylglycerol, and Kaempferol with key targets including AKT1, TNF, ESR1, and PPARG. Through molecular docking simulations, we observed distinct binding affinities and hydrogen bonding patterns that underscore the potential therapeutic efficacy of *D. alata* constituents against MDs. Notably, Alatanin demonstrated strong binding with AKT1 and PPARG, while Diosgenin exhibited notable interactions with TNF, suggesting their potential as effective therapeutic agents. These results elucidate the molecular basis by which bioactive compounds from *D. alata* may modulate key targets involved in menstrual health, providing a rationale for further experimental validation and clinical investigation. The comprehensive analysis of ligand-protein interactions enhances our understanding of *D. alata*'s pharmacological potential in managing MDs, warranting future studies to validate these computational findings in clinical trials.

**Fig. 7:**
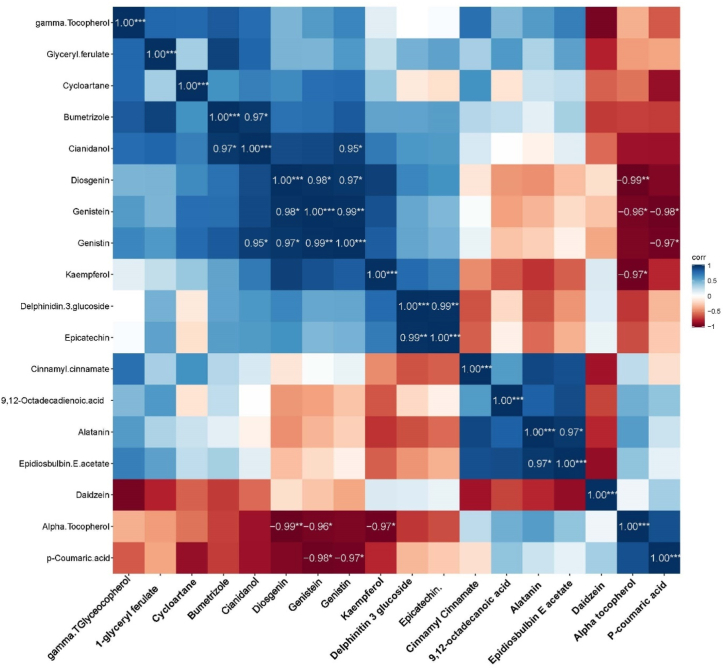
Correlation between the binding affinity/energy of molecules responsible for Menstrual problems in women.

**Fig. 8:**
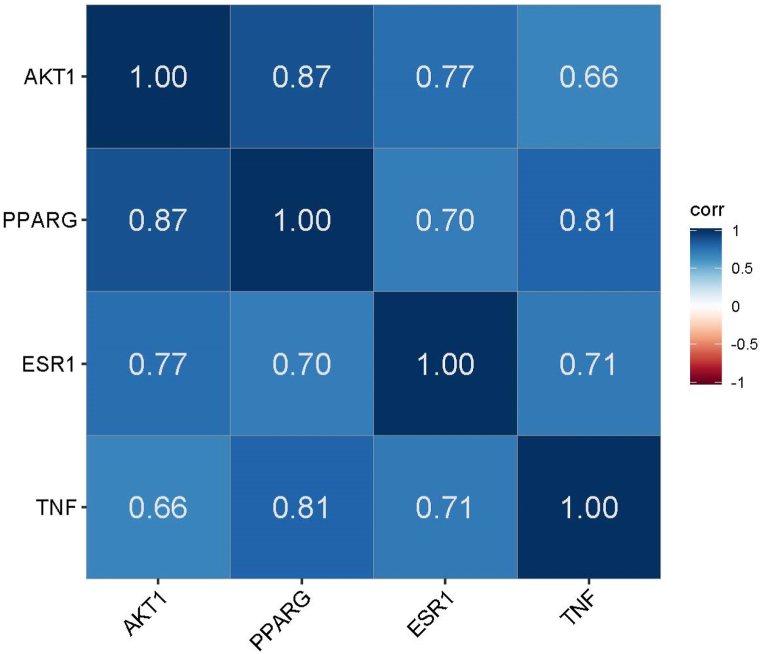
Correlation between the binding affinity/energy of selected hub genes responsible for Menstrual problems in women.

Fig. 7 revealed that the compounds like Bumetrizole vs cianidanol (0.97∗∗∗), Cianidanol vs Genistin(0.95∗∗∗), Diosgenin Vs Genistin(0.95∗∗∗), alatanin vs Epidiosbulbin E acetate (0.97∗∗∗) and Delpinitin-3-glucoside Vs Epicatechin (0.99∗∗∗), showed the maximum positive correlation. Likewise, Diosgenin vs Alpha tocopherol (0.99∗∗∗), Genistein Vs P-Coumaric acid (0.98∗∗∗), Genistin Vs P-Coumaric acid (0.97∗∗∗), Kaempferol vs Alpha tocopherol (0.97∗∗∗) and Genistein Vs Alpha tocopherol (0.96∗∗∗) showed the highest negative correlation. Remaining, combinations didn't show any positive and negative relationship. Similarly, correlation between hub genes showed maximum correlation with each gene (Fig. 8). Significantly, PPARG vs AKT1 showed maximum correlation (0.88∗∗∗), PPARG vs TNF (0.81∗∗∗), ESR1 vs AKT (0.77∗∗∗), ESR1 vs PPARG (0.70∗∗), ESR1 Vs TNF (0.71∗∗), and AKT1 vs TNF (0.81∗∗∗), negative relationship was not noticed in this correlation study.

### 3.6 Estrogen receptor alpha (ESR1)

**Fig. 9:**
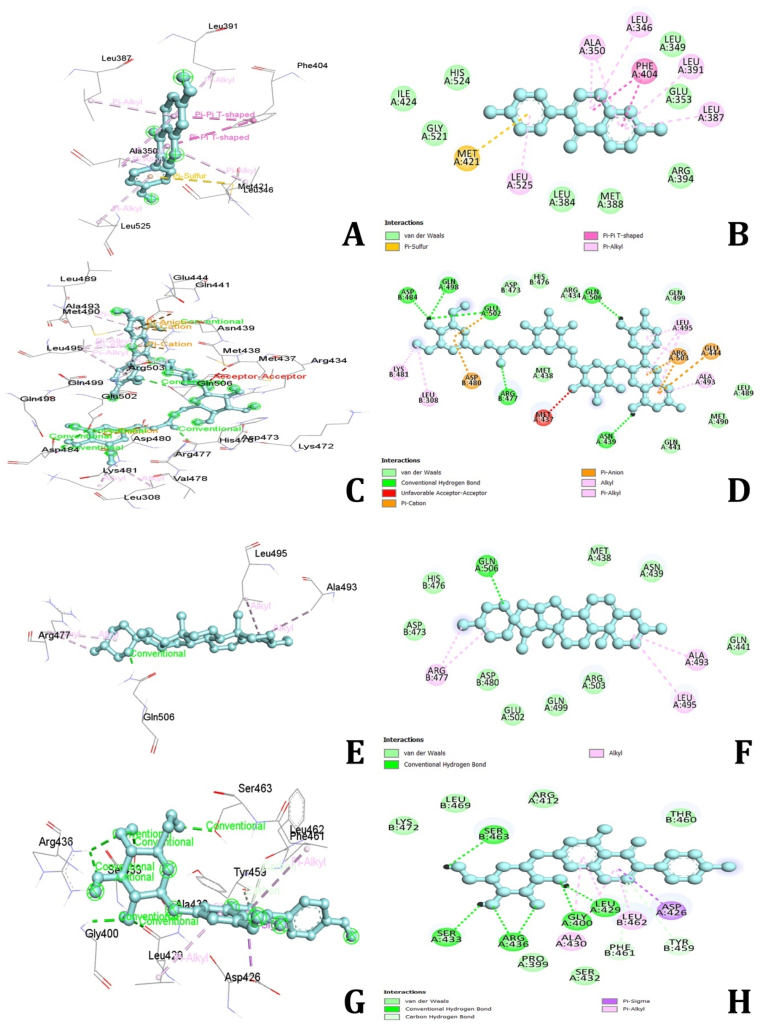
2D and 3D interactions of amino acids of Estrogen receptor 1 with Alatanin(A&B), Epidiosbulbin E acetate (C&D), Genistin (E&F) and Diosgenin (G&H).

The molecular docking simulations revealed that several bioactive compounds from *D. alata* exhibit strong binding affinities with the ESR1 gene. The compounds with the highest binding affinities were Daidzein (−9.2 kcal/mol), Alatanin (−9.0 kcal/mol), Diosgenin (−8.9 kcal/mol), Genistin (−8.6 kcal/mol), and Epicatechin (−8.5 kcal/mol). Compounds with binding energies below −8.5 kcal/mol were considered to have moderate binding affinities. **Daidzein** showed the highest binding affinity with ESR1, forming hydrophobic Pi-Pi T-shaped and Pi-Alkyl bonds with residues PHE404, LEU346, ALA350, and LEU387, and a Pi-Sulfur bond with MET421 (Fig. 9A and B). **Alatanin** exhibited hydrogen bonding interactions with ASP484, GLN498, GLU502, GLN506, ARG477, and ASN. Additionally, it formed hydrophobic Alkyl and Pi-Alkyl bonds with LYS481, LEU308, ALA493, LEU495, and LEU439, as well as electrostatic Pi-cation and Pi-anion bonds with ASP480, ARG503, and GLU444 (Fig. 9C and D). **Diosgenin** interacted through hydrogen bonds with GLN506 and hydrophobic Alkyl bonds with ARG477, ALA493, and LEU495 (Fig. 9E and F). **Genistin** formed hydrogen bonds with GLY400, ARG436, SER463, SER433, TYR459, and PHE461, and hydrophobic Alkyl bonds with ARG477, ALA493, and LEU495. **Epicatechin** showed hydrogen bonding interactions with ARG394 and PHE404, along with hydrophobic Pi-Alkyl bonds involving LEU384, LEU349, ALA350, LEU387, and LEU391 (Fig. 9G and H). The remaining compounds demonstrated moderate binding energies and formed various types of interactions, including hydrogen bonds, hydrophobic interactions, and electrostatic interactions. These findings suggest that the bioactive compounds in *D. alata* interact strongly with ESR1, highlighting their potential therapeutic roles in treating MDs. Further experimental validation is required to confirm these computational predictions and explore their clinical relevance.

### 3.7 Tumor necrosis factor alpha (TNFα) receptor

**Fig. 10:**
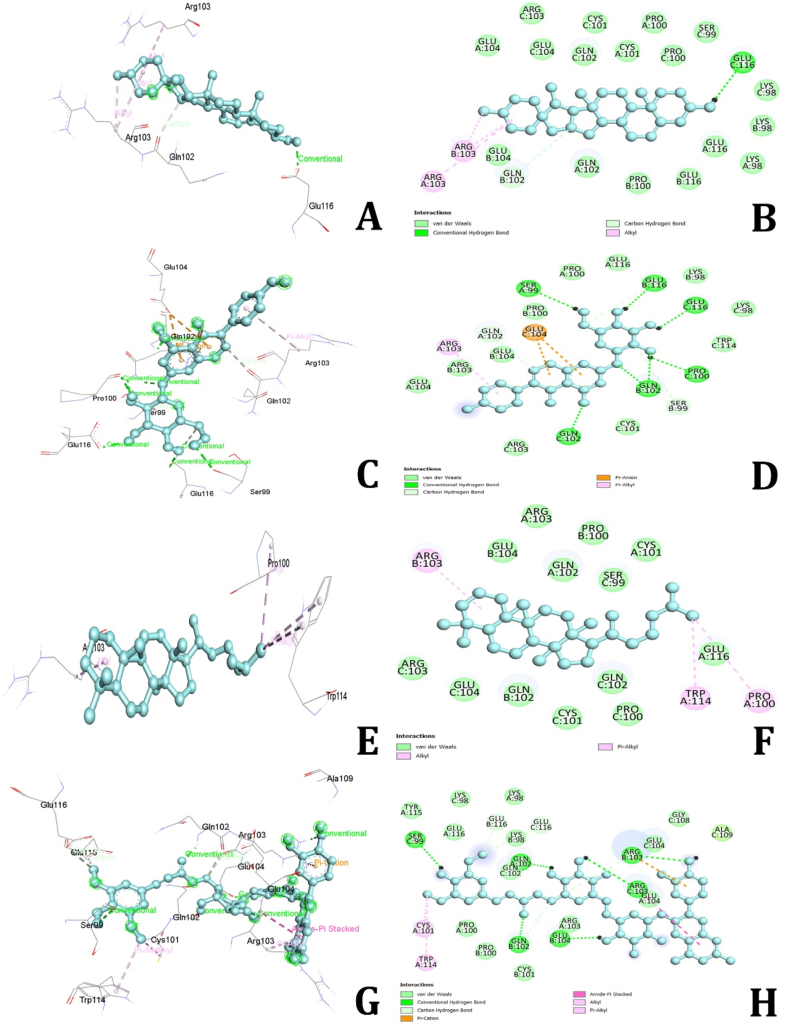
2D and 3D interactions of amino acids of Tumour Necrosis Factor Alpha receptor with Diosgenin (A&B), Genistin (C&D), Cycloartane (E&F) and Kaemferol (G&H).

The molecular docking simulations identified several bioactive compounds from *Dioscorea alata* that exhibit strong binding affinities with the TNFα receptor. The compounds with the highest binding affinities included Diosgenin (−10.8 kcal/mol), Genistin (−10.7 kcal/mol), Cycloartane (−9.5 kcal/mol), Alatanin (−9.1 kcal/mol), Kaempferol (−9.1 kcal/mol), Bumetrizole (−9.1 kcal/mol), and Cianidanol (−9.1 kcal/mol). Compounds with binding energies below −9.0 kcal/mol were considered to have moderate binding affinities. Diosgenin displayed the strongest binding affinity with TNFα, forming hydrogen bonds with GLU116 and GLN102, as well as hydrophobic Alkyl bonds with ARG103 (Fig. 10A and B). Genistin interacted with TNFα through hydrogen bonds with GLN102, SER99, and GLU116, hydrophobic Alkyl bonds with ARG103, and an electrostatic Pi-anion bond with GLU104 (Fig. 10C and D). Cycloartane formed hydrophobic bond interactions with ARG103, PRO100, and TRP114. Alatanin exhibited hydrogen bonding interactions with GLN102, ARG103, SER99, GLU104, and GLU116. Additionally, it formed hydrophobic amide-Pi stacked Alkyl and Pi-Alkyl bonds with CYS101, TRP114, and ARG103, along with an electrostatic Pi-cation bond interaction with ARG103 (Fig. 10E and F). Kaempferol showed hydrogen bonding interactions with GLN102, GLU116, and SER99, and an electrostatic Pi-anion bond interaction with GLU116 (Fig. 10G and H). Bumetrizole formed hydrophobic amide and Alkyl bond interactions with LYS158, PHE161, VAL164, and LYS179, along with electrostatic Pi-anion and Pi-cation bond interactions with LYS179 and ASP292. Cianidanol exhibited hydrogen bonding interactions with GLU278, LEU156, and THR291, hydrophobic Pi-Alkyl bond interactions with VAL164, ALA177, and ALA230, and a Pi-Sulfur interaction with MET281. The remaining molecules demonstrated moderate binding energies and formed various types of interactions, including hydrogen bonds, hydrophobic interactions, and electrostatic interactions. These findings suggest that the bioactive compounds in *D. alata* interact effectively with TNFα, highlighting their potential therapeutic roles in managing MDs. Further experimental validation is needed to confirm these computational predictions and explore their clinical implications.

### 3.8 Peroxisome proliferator activated receptor gamma (PPARG)

**Fig. 11:**
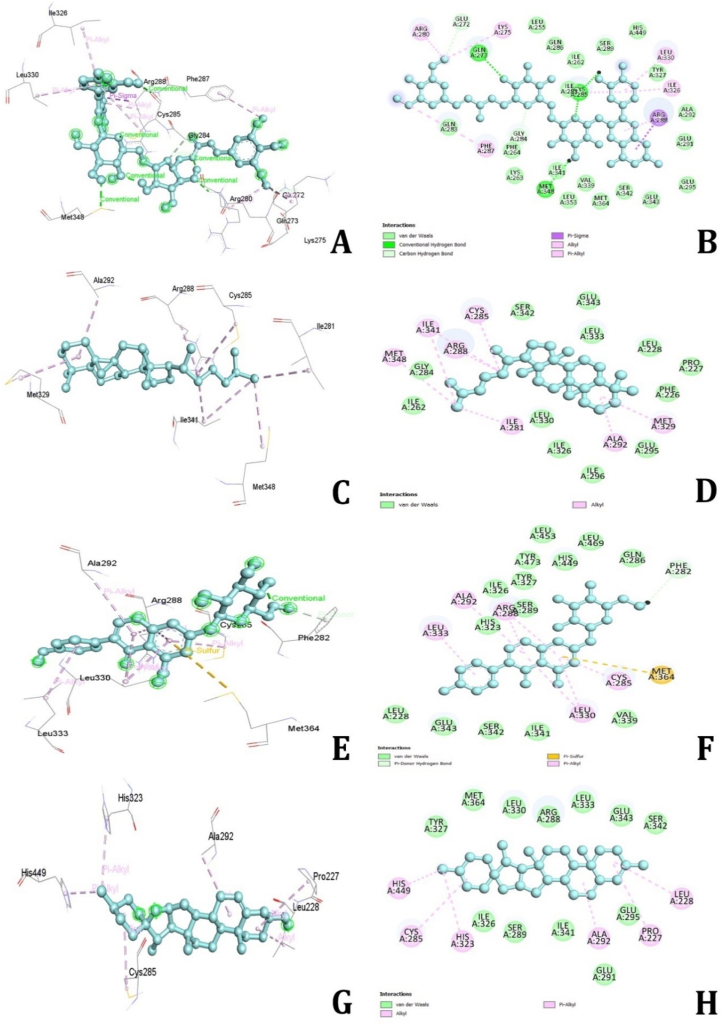
2D and 3D interactions of amino acids of PPARG receptor with Alatanin (A&B), Cycloartane (C&D), Genistin (E&F) and Diosgenin (G&H).

The molecular docking simulations revealed that several bioactive compounds from *D. alata* demonstrated strong binding affinities with the PPARG. The top compounds included Alatanin (−10.4 kcal/mol), Cycloartane (−9.9 kcal/mol), Genistin (−9.5 kcal/mol), Epidiosbulbin E acetate (−9.3 kcal/mol), and Diosgenin (−9.3 kcal/mol), while other molecules exhibited binding energies less than −9.0 kcal/mol. **Alatanin** showed robust binding with PPARG through hydrogen bonds with residues such as GLN273, CYS285, MET348, GLU272, and GLY284. Additionally, it formed hydrophobic Pi-Sigma, Pi-Alkyl, and Alkyl bond interactions with ARG288, ILE326, LEU330, LYS275, and ARG280 (Fig. 11A and B). **Cycloartane** interacted primarily via hydrophobic Alkyl bonds with residues MET348, ILE341, ARG288, CYS285, ILE281, ALA292, and MET329 (Fig. 11C and D). **Genistin** exhibited hydrogen bond interactions with ARG288 and SER342, along with hydrophobic Pi-Sigma and Pi-Alkyl bonds with CYS285, VAL339, and ALA292 (Fig. 11E and F). **Epidiosbulbin E acetate** formed hydrophobic Pi-Sigma, Pi-Alkyl, and Alkyl bond interactions with ILE341, LEU333, ARG288, CYS285, and VAL339 (Fig. 11G and H). **Diosgenin** displayed hydrophobic Pi-Alkyl and Alkyl bond interactions with residues HYS449, CYS285, HYS323, ALA229, PRO227, and LEU288. Other molecules demonstrated moderate binding energies and various types of interactions, including hydrogen bonds, hydrophobic interactions, and electrostatic interactions. These findings indicate that the bioactive compounds in *D. alata* have significant potential for therapeutic application in targeting PPARG, which may contribute to their efficacy in treating MDs. Further experimental validation is warranted to confirm these computational predictions and explore their clinical relevance.

### 3.9 AKT serine/threonine kinase 1 (AKT1)

**Fig. 12:**
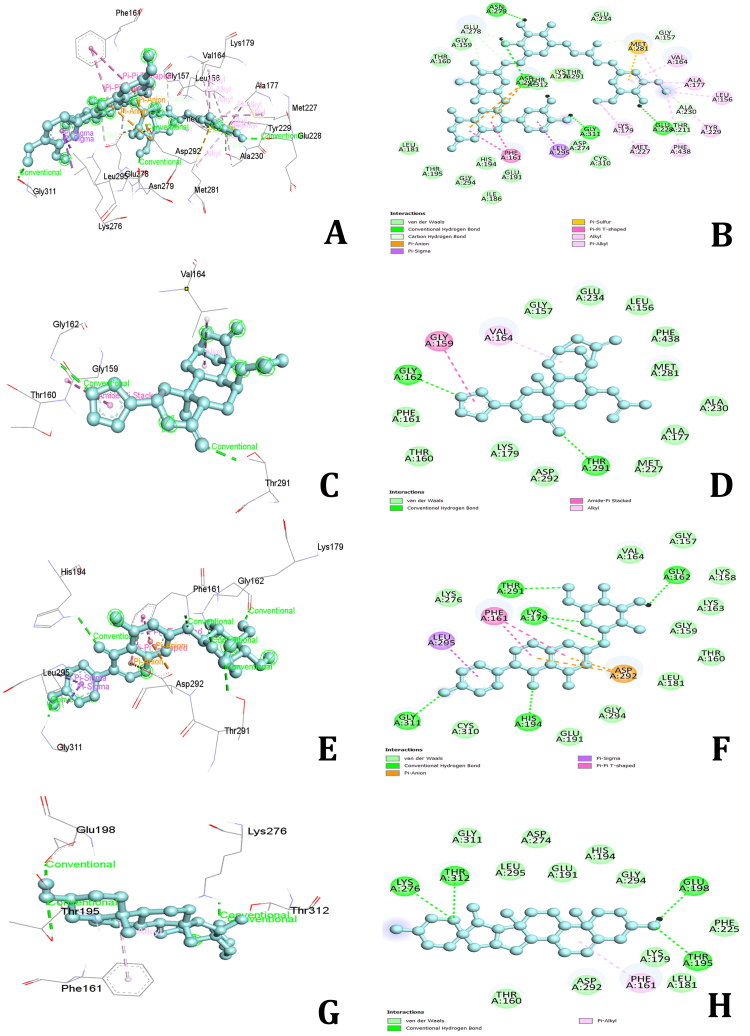
2D and 3D interactions of amino acids of AKT Serine/Threonine Kinase 1 (AKT1) receptor with Daidzein (A&B), Alatanin (C&D), Diosgenin (E&F) and Genistin (G&H).

The AKT1 receptor exhibited the highest binding affinity with several compounds from *Dioscorea alata*. Notably, Alatanin showed the strongest binding affinity (−10.5 kcal/mol), followed by Epidiosbulbin E acetate (−9.7 kcal/mol), Genistin (−8.9 kcal/mol), Diosgenin (−8.8 kcal/mol), Bumetrizole (−8.7 kcal/mol), Epicatechin (−8.6 kcal/mol), Alpha-tocopherol (−8.6 kcal/mol), and Cianidanol (−8.1 kcal/mol). Other compounds had binding energies of less than −8.0 kcal/mol. Alatanin exhibited numerous interactions, including hydrogen bonds with residues GLU228, ASN279, ASP292, GLY311, GLY157, GLU278, and ALA230. It also showed hydrophobic Pi-Sigma interactions with LEU295 and Pi-Pi T-shaped interactions with THE161, ALA177, ALA230, LEU156, MET281, MET227, LYS179, TYR229, PHE438, VAL164, and ALA177. Additionally, Alatanin formed electrostatic Pi-anion bonds with ASP292 and Pi-sulfur bonds with MET281 (Fig. 12A and B). Epidiosbulbin E acetate had hydrogen bond interactions with GLY162 and THR291, and hydrophobic amide and alkyl bond interactions with GLY159, THR160, and VAL164 (Fig. 12C and D). Genistin interacted through hydrogen bonds with LYS179, HIS194, THR291, GLY311, and GLY162. It also formed Pi-Sigma and Pi-Pi T-shaped hydrophobic bonds with LEU295 and PHE161, along with Pi-anion bonds with ASP292 (Fig. 12E and F). Diosgenin exhibited hydrogen bond interactions with THR195, LYS276, THR312, and GLU198. It also had hydrophobic Pi-alkyl bonds with THE161 and electrostatic Pi-cation bonds with ARG103 (Fig. 12G and H). Other compounds such as Bumetrizole, Epicatechin, and Alpha-tocopherol showed various interactions. Bumetrizole had hydrophobic amide and alkyl bonds with LYS158, PHE161, VAL164, and electrostatic Pi-anion and Pi-cation bonds with LYS179 and ASP292. Epicatechin had hydrogen bonds with ALA230 and hydrophobic Pi-Sigma interactions with MET28a, and Alpha-tocopherol had hydrogen bonds with GLU228 and hydrophobic alkyl bonds with VAL164, LEU181, PHE161, and ALA177. These interactions highlight the potential of *D. alata* compounds in targeting AKT1, suggesting their therapeutic efficacy in related treatments.

Furthermore, hydrogen bonding between a compound and its receptor protein is a crucial interaction for biological macromolecules, as it stabilizes protein structures and specific protein-ligand interactions. Our findings indicate that the majority of amino acid residues involved in these interactions—including hydrophobic, electrostatic, and other types of bonds—play a significant role in stabilizing the complexes within the receptor sites related to women's MDs. These interactions facilitate the compounds' deep embedding into the active site pockets, forming tight binding complexes. This analysis lays the groundwork for future studies aimed at identifying and isolating these bioactive compounds, potentially leading to the development of novel therapeutics for treating various menstrual-related issues in women.

## 4 Discussion

MDs significantly affect women's quality of life due to the associated short- or long-term discomfort [56]. Increasing evidence suggests that multi-component therapies, which involve the simultaneous effects of multiple agents on various targets, may be effective in managing complex conditions [57]. Traditional medicine often addresses diseases by balancing the body's overall bias, typically using prescriptions that include several herbal plants and components [58]. Herbs play a crucial role in the nutritional food chain in traditional medicine, providing nearly all necessary minerals and organic elements directly or indirectly. Medicinal and food plants contain numerous phytomolecules that can treat various health issues [8,59]. However, the complexity of bioactive compounds in plants poses challenges for traditional research methodologies to fully elucidate their mechanisms of action. Network pharmacology offers a valuable complement to traditional medicine and food research by integrating the ancient concepts of traditional/ethnic foods with contemporary scientific approaches [60,61]. In this study, we used network pharmacology and *in silico* analysis to explore the molecular pathways underlying the therapeutic effects of *D. alata* in the treatment of MDs, paving the way for future research and clinical applications.

*D. alata* L., commonly known as greater yam, is widely recognized for its health benefits, particularly in improving outcomes for postmenopausal women [61]. The tuber of *D. alata* contains a variety of bioactive molecules, including quercetin, kaempferol, ferulic acid, sinapic acid, caffeic acid, p-coumaric acid, (+)-catechin, Q9 chromene, γ-tocopherol-9, RRR-α-tocopherol, coenzyme Q9, cycloartane, 1-feruloylglycerol, cyanidin-3-monoglucoside, gallocatechin, epigallocatechin, myricetin, cyanidin 3-gentiobioside, alatanin C, genistin, daidzein, genistein, cyanidin 3-ferulyl gentiobioside, cyanidin 3-sinapylgentiobioside, peonidin 3-gentiobioside, alatanin 2, epicatechin-gallate, and vanillic acid [15,26,62]. Cheng et al. [63] demonstrated that compounds such as hydro-Q9 chromene, γ-tocopherol-9, RRR-α-tocopherol, coenzyme Q9, cycloartane, and 1-feruloylglycerol possess phenolic hydroxyl groups similar to phytoestrogens and exhibit antioxidant properties. Additionally, the estrogenic activity of these compounds was evaluated using ligand-dependent transcription activation assays. This highlights the potential of *D. alata* as a source of bioactive compounds with significant health benefits, particularly for women's health.

The bioactive compounds in *D. alata* exhibit significant hypoglycemic and antioxidant properties, which contribute to weight loss and the prevention of diabetes and obesity with minimal side effects [[64], [65], [66]]. Childhood and adolescent obesity are linked to numerous negative health outcomes. Hormones such as leptin, kisspeptin, and insulin, along with their effects on the hypothalamic-pituitary-ovarian axis, are associated with childhood obesity and early puberty. In adolescent girls and young women, obesity is related to premenstrual syndrome, premenstrual dysphoric disorder, dysmenorrhea, increased menstrual cycle irregularity, and polycystic ovarian syndrome (PCOS) [67]. Additionally, compounds such as alatanins A, B, and C, γ-tocopherol-9, α-tocopherol, coenzyme Q, 1-feruloylglycerol, cyanidin-3-glucoside, peonidin-3-gentiobioside, and hydro-Q chromene from *D. alata* have been shown to have beneficial effects on obesity [65]. In the present study, network pharmacology was utilized to predict the potential targets responsible for the effects of *D. alata* bioactive compounds on MDs, aiming to elucidate the molecular mechanisms involved. The analysis revealed several highly connected targets within the MDs Target PPI Network, including TNF, PPARG, AKT1, ESR1, MAPK14, MDM2, PTPN1, PGR, ESR2, MAPK1, EGFR, IL2, STAT3, KDR, MMP2, HDAC1, CYP1B1, OXTR, CYP26A1, SHBG, and ESRRA. Among these, PPARG, AKT1, ESR1, and TNF were identified as the top four primary targets mediating the effects of *D. alata* bioactive molecules. This finding aligns with Rani et al. [68], who found that the biomolecules in Saptasaram kashayam target proteins such as STAT3, ESR1, AKT1, and SHBG, which are implicated in MDs ers through PPI network interactions. Similarly, Li et al. [69] found that TNF and PPARG are key proteins involved in MDs in their network pharmacological study of Yuzhi Zhixue granule, a traditional Chinese medicine used to treat ovulatory dysfunctional uterine bleeding. These results corroborate our findings.

*D. alata* contains numerous chemical components that contribute to its wide range of biological activities, including the regulation and treatment of various illnesses and conditions [15]. In this study, we examined compounds such as Alatanin, Epidiosbulbin E acetate, Genistin, Diosgenin, Bumetrizole, γ-Tocopherol, Epicatechin, Cianidanol, Daidzein, Cinnamyl cinnamate, Kaempferol, and Cycloartane. Among these, specific compounds like Diosgenin, Alatanin, Cycloartane, and Daidzein were identified as potential contributors to the therapeutic effects on MDs.

Diosgenin holds promise as a therapeutic agent for various chronic disorders, including cancers such as breast cancer and chronic myeloid leukemia, and exhibits estrogenic activity. Its anti-inflammatory and neuroprotective actions are notable, particularly through suppression of NO, IL-1β, IL-6, protein kinase A (PKA), Akt, cPLA2, cAMP, and MAPK signaling pathways [66]. Diosgenin and its metabolites influence reproductive and non-reproductive processes via multiple signaling pathways, impacting proliferation, apoptosis, hormone secretion, and growth factors in both healthy and malignant ovarian cells, as well as uterine contractions. While Diosgenin shows promise in ovarian folliculogenesis, oogenesis, and fecundity, further validation through animal and clinical trials is warranted [67].

Alatanin, an anthocyanin molecule, has demonstrated estrogenic activity [70]Previous clinical trials with alpha and gamma-tocopherol have indicated that these compounds can effectively manage specific premenstrual syndrome symptoms, likely due to their effects on the central nervous system or their ability to alter peripheral endocrine function, thereby modifying serum concentrations of steroid hormones [71]. Cycloartane, a triterpenoid compound, has been shown to inhibit the growth of MDA-MB48 and MCF-7 breast cancer cell lines, indicating its potential role in preventing breast cancer in women [72]. Additionally, cycloartane's inhibition of 11β-hydroxysteroid dehydrogenases (11β-HSD1 and 11β-HSD2) suggests its potential as a novel treatment for excessive menstrual bleeding [73]. Similarly, daidzein (7,4′-dihydroxyisoflavone), a phenolic compound primarily derived from legumes, has garnered attention for its positive effects on women's reproductive health. Administering 50 mg/kg/day of daidzein to female Wistar rats has been shown to mitigate significant ovarian damage by reducing levels of IL-6, TNF-α, and free radicals, thus addressing fertility decline caused by PCOS [74]. This is consistent with our findings, as daidzein exhibited the highest binding affinity among the proteins we examined. In addition, daidzein has been demonstrated to be effective in treating inflammatory illnesses. Daidzein substantially decreased the growth and size of ovarian cancer tumors by blocking the RAF/MEK/ERK signaling mechanism, G2/M2 cell cycle arrest, and reducing p-FAK, p-PI3K, p-AKT, p-GSK3β, p21 or cyclin D1 expression in ovarian cancer cells (Singla et al., 2024)

Khezri et al. [75] reported that administering genistein to rats with PCOS significantly increased gonadotropin and steroid hormone production while reducing insulin resistance. This resulted in improved follicular growth and hormone balance in the ovarian tissue of PCOS rats. Additionally, previous studies have shown that genistein regulates insulin and glucose metabolism, aiding in the treatment of diabetes in postmenopausal women and diabetic animal models [[76], [77], [78]]. Cianidanol has also been identified as a selective agonist of the beta estrogen receptor in PCOS [79,80]. Epicatechin, a naturally occurring polyphenol widely found in plants, regulates various biological processes and signaling pathways. It is one of the most abundant polyphenol compounds in the human diet. Recent studies have highlighted epicatechin's potential as a promising therapy for Premature Ovarian Insufficiency (POI), as it can down-regulate increased oxidative stress levels via the PI3K/AKT/Nrf2 signaling pathway [81].

Diosgenin, a bioactive molecule abundant in *D. alata*, serves as a precursor for manufacturing numerous steroidal drugs in the pharmaceutical industry and exhibits possible estrogenic activity. Research indicates that diosgenin improves ovarian function and embryo quality in women undergoing *in vitro* oocyte maturation and fertilization when included in a multinutrient supplement [82]. Moreover, older mice treated with diosgenin showed higher blood levels of anti-Mullerian hormone and an increased number of primary ovarian follicles, which helped prevent reproductive aging [83]. Luo et al. [84] identified kaempferol as a bioactive compound with significant progestogenic effects in a murine model and anti-proliferative effects in ovarian cancer cells. Kaempferol also enhances the expression of ZBTB16 and FKBP5 in the uterus [85]. Additionally, it is utilized to manage symptoms such as irregular premenopausal bleeding or increased uterine growth in conditions like endometriosis. Kaempferol treatment has been shown to downregulate several genes associated with polycystic ovarian syndrome [86].

Furthermore, genes in Fig. 2a and b, also involved in the regulation of menstrual cycle. For instance, Protein Tyrosine Phosphatase Non-Receptor Type 1 (PTPN1) that may contribute to the pathophysiology of PCOS [87]. Disruptions in the PGR pathway can lead to conditions like endometriosis and irregular menstruation [88]. Interruptions in EGFR signaling have been linked to menstrual disorders, notably abnormal vaginal bleeding [89]. IL-2 may also play a role in menstrual disorders, particularly in conditions like endometriosis and premenstrual syndrome. For instance, IL2 level decline during the luteal phase of the menstrual cycle in healthy women. This decrease may contribute to premenstrual immune suppression, potentially increasing susceptibility to infections [90,91]. (STAT3) is a transcription factor integral to various cellular processes, including proliferation, differentiation, and apoptosis. Its role in reproductive health is underscored by its involvement in menstrual disorders, particularly endometriosis. Significantly, STAT3 regulates the formation of slit-like structures in the uterine lumen, while stromal STAT3 suppresses proliferative activity, coordinating uterine receptivity and function [92]. KDR is crucial for normal endometrial function and menstrual cycle regulation. Alterations in KDR expression or function have been linked to various menstrual disorders [93]. Matrix metalloproteinase-2 (MMP-2), also known as gelatinase A, is an enzyme that plays a crucial role in the remodeling of the extracellular matrix (ECM) during the menstrual cycle. MMP-2 is involved in the degradation of ECM components, allowing for the necessary tissue remodeling. Significantly, human endometrial stromal cells produce proteinases, including MMP-2, in response to progesterone withdrawal, a physiological stimulus for menstruation [94]. Expression of HDAC1 is significantly elevated in endometriotic tissues compared to normal endometrial tissues. This overexpression correlates with the expression of estrogen and progesterone hormone receptors, suggesting that HDAC1 may influence the hormonal responsiveness of endometriotic lesions [95]. Cytochrome P450 1B1 (CYP1B1) is an enzyme involved in the metabolism of estrogens, converting them into metabolites that can influence estrogen receptor activity. Significantly, certain variants of the CYP1B1 gene may influence the onset of menopause in Chinese women, potentially affecting estrogen metabolism and reproductive aging [96]. The oxytocin receptor (OXTR) plays a significant role in various reproductive processes, including the regulation of the menstrual cycle. Significantly, disruptions in oxytocin pathways may contribute to menstrual irregularities [97] likewise epigenetic dynamics of the OXTR gene across the menstrual cycle indicates that OXTR methylation is dynamic in naturally cycling women but relatively stable in women on birth control, implying a potential link between OXTR expression and menstrual function [98]. Cytochrome P450 26A1 (CYP26A1) is an enzyme responsible for the metabolism of retinoic acid, that plays a crucial role in cellular differentiation and proliferation within the endometrium. For instance, CYP26A1 expression is significantly down-regulated in endometriotic tissues compared to normal endometrium, suggesting a disruption in retinoic acid metabolism. This alteration may contribute to the pathogenesis of endometriosis by affecting endometrial cell growth and differentiation [99].

Gene Ontology (GO) annotation and KEGG pathway enrichment analyses revealed that the primary pathways affected by *D. alata* compounds were involved in ovarian follicle formation, hormone stimulation, extracellular stimuli, steroid hormone receptor signaling, and the intracellular estrogen receptor signaling pathway. KEGG pathway analysis indicated that the suggesting that *D. alata* treats MDs by influencing these signaling pathways. These pathways play critical roles in the physiological and psychological functions of the female reproductive system, including the menstrual cycle [18,100]. In addition, compounds from *D. alata* stimulate human alpha and beta estrogen receptors [63].

Analysis of upstream pathway activity revealed that estrogen, TNF-α, MAPK/ERK, and PI3K/Akt are pertinent to the hormonal and immunological responses associated with menstrual illnesses such as endometriosis, polycystic ovarian syndrome (PCOS), and menorrhagia. The function of estrogen signaling is crucial in regulating the menstrual cycle, emphasizing the constant up-regulation of this pathway in the data would underscore its direct significance to menstrual health [101]. The current results have important implications regarding diseases such as endometriosis, which is characterized by widespread inflammation and immunological dysregulation. The TNF-α pathway, in particular, promotes inflammatory responses and contributes to the course of the disease. Additionally, the aberrant proliferation of cells observed in polycystic ovary syndrome (PCOS) and other menstruation-related diseases is associated with the MAPK/ERK and PI3K/Akt pathways, which are critical for cell survival and growth. Therefore, it can be inferred that the pathophysiology of these diseases is directly influenced by estrogen signaling when these pathways are upregulated. In menstrual diseases, the pathways that are down-regulated, like TGF-β and Hippo, are important because they regulate the immune system, aid in cell proliferation, and heal damaged tissues. Studies show that the TGF-β superfamily genes are downregulated during menstruation, leading to an imbalance between molecules that promote inflammation and those that regulate it. Problems like primary dysmenorrhea, in which abnormalities in the expression of these pathways make periods more painful, have been associated to this discord [102].

Komesaroff et al. [103] highlighted diosgenin, a steroid hormone precursor, for its weak estrogen-binding activity and its role in cholesterol transport. Diosgenin has demonstrated potential pro- and antineoplastic properties. Dioscorea products are widely promoted for their purported benefits, such as alleviating menopausal symptoms. Hsu et al. [18] conducted a two-center, randomized, double-blind, placebo-controlled clinical trial in Taiwanese women and found that *D. alata* extracts may be beneficial for menopausal issues, particularly in improving psychological parameters. Our current study corroborates these findings, showing that *D. alata* compounds have therapeutic benefits for women's menstrual problems.

Using topological analysis, four key genes—ESR1, TNF, AKT1, and PPARG—emerged as central to our investigation. PPARG, a ligand-activated transcription factor, plays critical roles in adipocyte development, insulin sensitivity, lipid metabolism, and atherosclerosis in tissues such as adipose, macrophages, intestines, and ovaries [104]. PPARG also holds promise as a potential regulator affecting PCOS risk and exhibits significant anti-inflammatory functions, partly through NF-κB pathway inhibition [105,106]. PPARs are actively expressed across the female reproductive system, governing gametogenesis, ovulation, corpus luteum regression, and embryo implantation [107,108]. Their reproductive function is primarily linked to their role in energy homeostasis, which has consequences for ovarian dysfunctions associated with obesity, dyslipidemia, hyperandrogenemia, and insulin resistance [109].

Ovarian hormones profoundly influence immune factors within the endometrium, including TNF-α and IL2, which are critical for infection prevention, endometrial preparation for implantation or menstruation, and subsequent repair. Dysregulation of the TNF-α pathway has been linked to conditions like uterine fibroids and endometriosis, potentially inducing apoptosis and cell dissociation in the endometrium, leading to menstrual shedding and heavy bleeding [110,111]. Several studies have reported vascular damage and endometrial bleeding following TNF-α administration. Furthermore, TNF may directly or indirectly stimulate apoptosis in endometrial epithelial and stromal cells during menstruation, and in the absence of implantation, it could facilitate the loss of endometrial tissue. Treatment of uterine epithelial cells with TNF induces growth arrest, death, and a disruption of epithelial cell–cell connections and vascular integrity [112].

ESR1, a ligand-activated nuclear receptor, plays a pivotal role in menstrual cycle timing and is associated with disorders such as premenstrual dysphoric disorder and PCOS [113,114]. In addition, ESR1, a key protein that mediates estrogen's protective effects, plays an essential role in both neuroprotection and menstrual health. In menopause, reduced estrogen levels, and consequently decreased ESR1 activity, lead to increased TTBK1 activity, contributing to tau hyperphosphorylation and Alzheimer's progression [41,115,116]. ESR1 binds steroid hormones and has been implicated in dysmenorrhea [117]. AKT1, a member of the serine-threonine protein kinases, is crucial for ovarian processes, including follicle stimulation, granulosa cell survival, and proliferation. It regulates cell survival, growth, metabolism, and differentiation through various downstream effectors and is frequently implicated in cancer proliferation and survival pathways, particularly in breast and ovarian cancers [118,119]. Additionally, AKT1 has been linked to progestin resistance in endometrial and breast cancers by reducing progesterone receptor expression [120].

PCOS is associated with three main risk factors: genetic predisposition, hormonal factors such as insulin resistance and hyperandrogenism, and environmental influences like nutrition and exposure to endocrine disruptors [121].Genes related to steroidogenesis and hormone effects, such as CYP11A1, CYP17, CYP19, and AR, are implicated in PCOS pathogenesis. Molecular docking analysis plays a critical role in predicting therapeutic targets for human ailments by assessing the binding energy of complexes. Our study revealed that *D. alata* compounds exhibited strong binding affinities to hub genes, suggesting they interact effectively within the active sites, forming stable complexes that regulate gene expression and activity in biological processes.

Collectively, *D. alata*'s diverse components act synergistically across multiple pathways, targeting numerous genes and proteins simultaneously to alleviate MDs. These findings underscore the potential of multi-component natural remedies in treating complex illnesses that often resist single-compound treatments. Nonetheless, our study has limitations, including uncertainties regarding bioavailability and the precise modulatory effects on suggested targets. Future research should focus on elucidating the mechanisms through which *D. alata* molecules effectively treat MDs, building on the groundwork laid by network pharmacology and *in silico* methodologies.

In conclusion, our investigation substantiates the use of *D. alata* products containing Cycloartane, Daidzein, Alatanin, and Diosgenin as promising treatments for menstrual problems. Continued research into their mechanisms of action will be crucial for advancing their therapeutic potential in clinical applications.

## 5 Conclusion

In conclusion, our study highlights the potential of *D. alata* bioactive compounds in the treatment of MDs. Through bioinformatics and network pharmacology, we systematically investigated the mechanisms underlying these compounds' actions. Specifically, our findings indicate that these bioactive compounds interact with key targets such as PPARG, TNF α, ESR1, and AKT1, influencing pathways critical to MDs. Moving forward, further *in vitro* and *in vivo* research is essential to establish the bioavailability and detailed mechanisms of action of *D. alata* bioactive compounds. It is important to note that our study was limited to network pharmacology and *in silico* molecular docking analysis. Nonetheless, the outcomes suggest that physiologically relevant components from *D. alata* could potentially alleviate women's menstrual diseases. This research provides a theoretical basis for considering *D. alata* as a viable and effective treatment approach for menstrual problems in clinical settings. Interestingly, despite its potential therapeutic benefits, *D. alata* remains underutilized globally, although it has been traditionally consumed as a staple by indigenous communities in various regions of India and neighbouring countries. By incorporating *D. alata* flour into fortified food products, we may offer a sustainable solution to women suffering from severe menstrual problems, enhancing their overall health and quality of life. Continued exploration and validation of these findings could pave the way for broader applications of *D. alata* in menstrual health management.

## CRediT authorship contribution statement

**Rajendran Silambarasan:** Methodology, Investigation, Formal analysis, Data curation. **A. Kasthuri Nair:** Validation, Project administration, Conceptualization. **Gomathi Maniyan:** Software, Resources, Formal analysis, Data curation. **R. Vijaya:** Validation, Resources. **Reshma V.R. Nair:** Methodology. **J. Hareendran Nair:** Project administration, Funding acquisition. **S. Nishanth Kumar:** Writing – review & editing, Writing – original draft. **Shan Sasidharan:** Resources, Project administration.

## Ethics approval and consent to participate

Not applicable.

## Data availability

All the data generated and analyzed during this investigation/study are included in this manuscript and the supplementary materials supplied.

## Funding statement

The authors have no relevant financial or nonfinancial interests to disclose.

## Declaration of competing interest

The authors declare the following financial interests/personal relationships which may be considered as potential competing interests: Shan Sasidharan reports financial support was provided by 10.13039/501100002332Semmelweis University. If there are other authors, they declare that they have no known competing financial interests or personal relationships that could have appeared to influence the work reported in this paper.
