# Supplementary material for: Exploring the molecular mechanism of Dioscorea alata L. for the treatment of menstrual disorders using network pharmacology and molecular docking
Source: Heliyon. 2025 Feb 8;11(4):e42582. doi: 10.1016/j.heliyon.2025.e42582 (PMC11870275; doi:10.1016/j.heliyon.2025.e42582)
Supplement: Multimedia component 1 [file mmc1.docx]

**Supp. Table 1.** Details of compounds selected from the *Dioscorea alata*

| **S.**  **No.** | **Name & Structure** | **Molecular Formula** | **Molecular weight** | **PubChem ID** |
| --- | --- | --- | --- | --- |
| 1 | Diosgenin  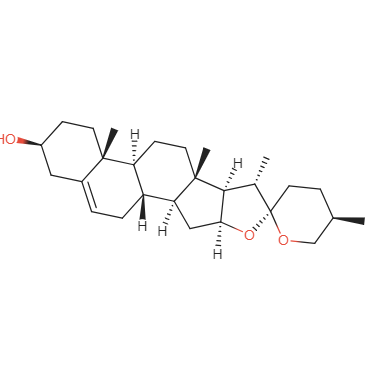 | C_27_H_42_O_3_ | 414.6 g/mol | 99474 |
| 2 | Delphinidin 3-glucoside chloride  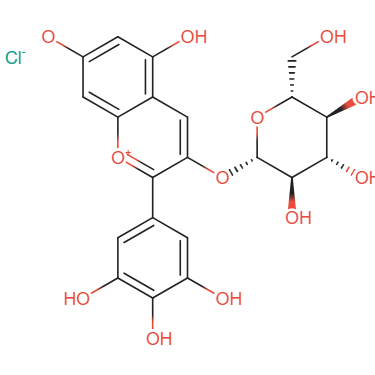 | [C_21_H_21_ClO_12_](https://pubchem.ncbi.nlm.nih.gov/#query=C21H21ClO12) | 500.8 g/mol | 165558 |
| 3 | Cinnamyl cinnamate  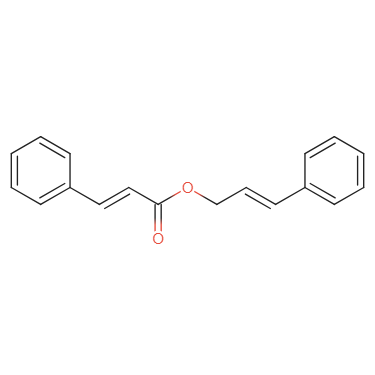 | C_18_H_16_O_2_ | 264.3 g/mol | 1550890 |
| 4 | Bumetrizole  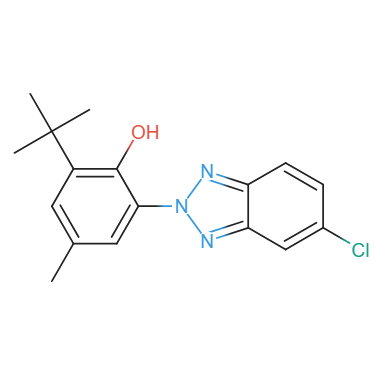 | C_17_H_18_ClN_3_O | 315.8 g/mol | 62531 |
| 5 | 9,12-Octadecadienoic acid  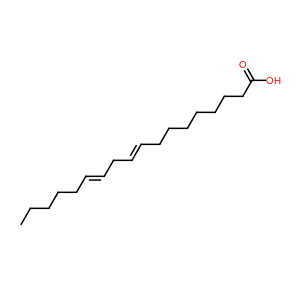 | C_18_H_32_O_2_ | 280.45 g/mol | 3931 |
| 6 | p-Coumaric acid  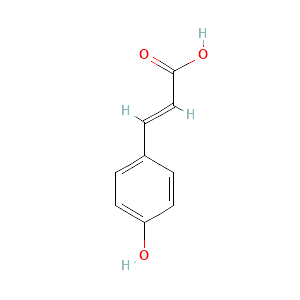 | C_9_H_8_O_3_ | 164.16 g/mol | 637542 |
| 7 | Kaempferol  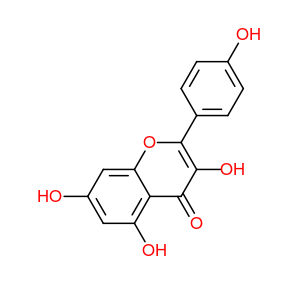 | C_15_H_10_O_6_ | 286.24 g/mol | 5280863 |
| 8 | 8-epidiosbulbin E ace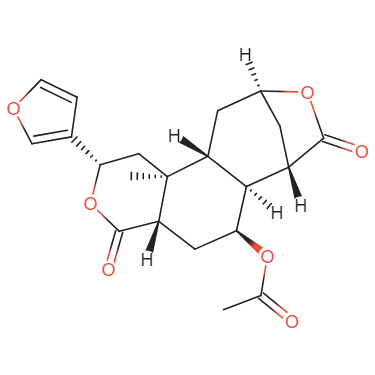 | C_21_H_24_O_7_ | 388.4 g/mol | 134715250 |
| 9 | 1-feruloylglycerol  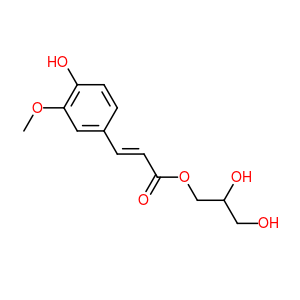 | C_13_H_16_O_6_ | 268.26 g/mol | 11311691 |
| 10 | Cycloartane  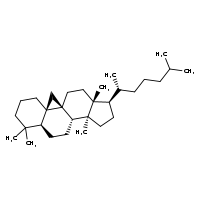 | C_30_H_52_ | 412.7 g/mol | 160497 |
| 11 | Alpha-Tocopherol  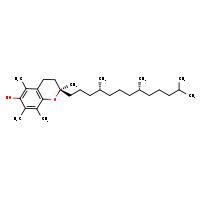 | C_29_H_50_O_2_ | 416.7 g/mol | 14985 |
| 12 | gamma-Tocopherol  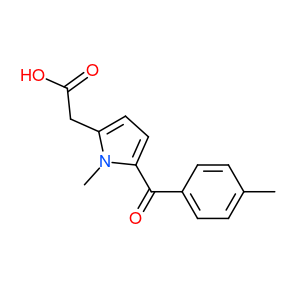 | C_28_H_48_O_2_ | 416.7 g/mol | 92729 |
| 13 | Catechin  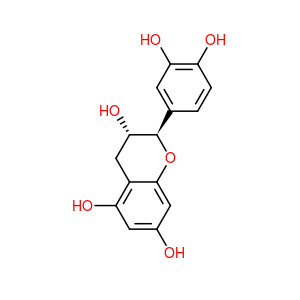 | C15H14O6 | 290.27 g/mol | 9064 |
| 14 | Epicatechin  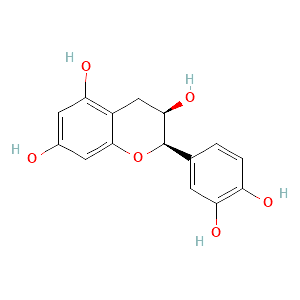 | C_15_H_14_O_6_ | 290.27 g/mol | 72276 |
| 15 | Alatanin 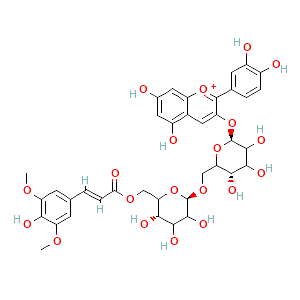 | C_38_H_41_O_20_^+^ | 817.7 g/mol | 44256751 |
| 16 | Genistein  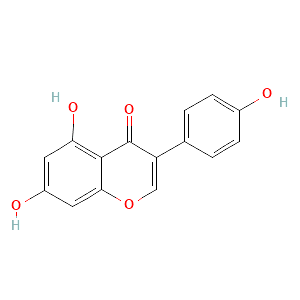 | C_15_H_10_O_5_ | 270.24 g/mol | 5280961 |
| 17 | Daidzein  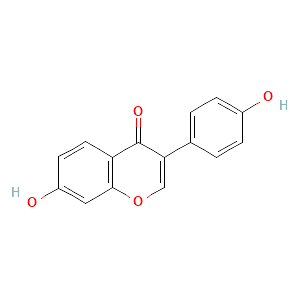 | C_15_H_10_O_4_ | 254.24 g/mol | 5281708 |
| 18 | Genistin  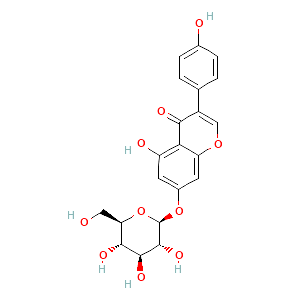 | C_21_H_20_O_10_ | 432.4 g/mol | 5281377 |
