# Supplementary material for: Exploring the molecular mechanism of Dioscorea alata L. for the treatment of menstrual disorders using network pharmacology and molecular docking
Source: Heliyon. 2025 Feb 8;11(4):e42582. doi: 10.1016/j.heliyon.2025.e42582 (PMC11870275; doi:10.1016/j.heliyon.2025.e42582)
Supplement: Multimedia component 9 [file mmc9.docx]

**Supp. Table 5.** Molecular docking analysis of phytochemicals with four different receptors and comparison with *D. alata* molecules

| **S.no** | **Receptor**  **name** | **Compound name** | **Binding affinity** | **Hydrogen bond** | **Hydrophobic bond** | **Electrostatic interaction** | **Others** |
| --- | --- | --- | --- | --- | --- | --- | --- |
| 1 | ESR1 | 9,12-Octadecadienoic acid | -5.4 | GLN506 | Alkyl bond: ALA 493, LEU 495, MET490, Leu 489 |  |  |
| 2 |  | Alatanin | -9 | ASP484, GLN498, GLU 502, GLN 506, ARG 477, ASN 439 | **Alkyl bond and Pi- alkyl bond:** LYS481, LEU 308, ALA 493, LEU 495 | **Pi-cation and Pi- anion bond:** ASP 480, ARG 503, GLU 444 |  |
| 3 |  | Alpha-Tocopherol | -8.4 | - | Alkyl bond: ALA 350, LEU 354, LEU 525, TRP 383, Leu 384 | - |  |
| 4 |  | Bumetrizole | -8 | SER 463, ALA 430, | **Alkyl, Pi-Alkyl and amide bond:** LEU 429, ALA430, LYS 472, LEU 469 | **Bi cation: LYS 472** | **Pi-sulfur:** MET 437 |
| 5 |  | Cianidanol | -7.4 | PHE 461, ARG436 | **Pi-alkyl bond:** ALA430, LEU 462, LEU 429.  **Pi-sigma bond:** ASP 426 |  |  |
| 6 |  | Cinnamyl cinnamate | -6.8 | - | Pi-sigma bond: VAL 376, ILE 358  Pi-Pi stacked and Pi-Alkyl bond: LEU 379, TRV 383 | - | - |
| 7 |  | **Cycloartane** | -7.2 |  | Alkyl and Pi-Alkyl bond: PHE367, LEU379, VAL376, ILE358, LYS362. |  |  |
| 8 |  | Daidzein | -9.2 | - | Pi-Pi T-shaped and Pi-Alkyl bond: PHE 404, LEU 346, ALA 350, LEU 387 | - | **PI-Sulfur bond:** MET 421 |
| 9 |  | Delphinidin 3-glucoside | -7.7 | GLY 400, ARG 436, PHE 461, Ser 463 | Pi-Alkyl bond: ALA430. |  |  |
| 10 |  | **Diosgenin** | -8.9 | GLN506, | Alkyl bond: ARG477, ALA493, LEU495. |  |  |
| 11 |  | Epicatechin | -8.5 | ARG394, PHE 404 | **Pi-Alkyl bond:** LEU 384, LEU 349, ALA 350, LEU 387, LEU 391 |  |  |
| 12 |  | Epidiosbulbin E acetate | -7.9 | ASP 374, SER 468 | Alkyl bond: LEU 461 |  |  |
| 13 |  | gamma-Tocopherol | -6.3 | VAL 368, ASP 369 | Alkyl bond: LEU 310, ALA 318, ARG 363, LEU 310. |  |  |
| 14 |  | Genistein | -7.8 | GLY400, SER 463, THR 460, ASP 426. | Pi-Alkyl and Amide-Pi Stacked: LEU 429, ALA 430. | Pi-Anion: ASP 426, |  |
| 15 |  | Genistin | -8.6 | GLY 400, ARG 436, SER 463, SER433, TYR 459, PHE461 | Pi-Sigma bond: ASP 426, Pi-Alkyl ALA 430, LEU 462, LEU 429, |  |  |
| 16 |  | Glyceryl ferulate | -5.9 | ALA 307, GLY 366, VAL 364, LYS 362, | Pi-Sigma and Alkyl  LEU 310, ALA318. |  |  |
| 17 |  | Kaempferol | -8.3 | ARG 394, PHE404, | Pi-Pi T-shaped and Pi-Alkyl PHE 404, LEU 384, MET 421, ILE 424, ALA350, LEU 387, LEU 391. |  |  |
| 18 |  | p-Coumaric acid | -6.3 |  | PHE 404, ALA 350, LEU 387, LEU 391, |  |  |
| 19 | TNF | 9,12-Octadecadienoic acid | -5.5 | SER99, GLN102 | ARG 103 |  |  |
| 20 |  | Alatanin | -9.1 | GLN 102, ARG 103, SER 99, GLU104, GLU116, | Amide-Pi Stacked Alkyl and Pi-Alkyl  CYS 101, TRP114, ARG 103. | Pi-Cation ARG103, |  |
| 21 |  | Alpha-Tocopherol | -7 | SER99 | ARG103 |  |  |
| 22 |  | Bumetrizole | -9.1 | PRO  100, GLN102, | Amide-Pi Stacked AND Alkyl PRO100, CYS 101, CYS 69, TRP 114. |  |  |
| 23 |  | Cianidanol | -9.1 | GLN102, GLU116, SER99, TYR115, |  | Pi-Anion: GLU116 |  |
| 24 |  | Cinnamyl cinnamate | -7.3 | GLN102, | Amide-Pi Stacked AND Alkyl PRO100, CYS 101 | Pi-Anion GLU116, |  |
| 25 |  | Cycloartane | -9.5 | - | ARG103, PRO100, TRP114 | - |  |
| 26 |  | Daidzein | -8.3 | GLU116, GLN102, GLU104 |  | Pi-anion: GLU116 |  |
| 27 |  | Delphinidin 3-glucoside | -7.8 | GLU104, GLN102, | Pi alkyl bond: ARG103 | Pi-cation and Pi-Anion: ARG103, GLU104, |  |
| 28 |  | Diosgenin | -10.8 | GLU116, GLN 102 | Alkyl bond: ARG 103 |  |  |
| 29 |  | Epicatechin | -9 | GLN102, ARG 103, GLU 116, |  | Pi-anion bond: GLU116 |  |
| 30 |  | Epidiosbulbin E acetate | -8.4 | SER 99, GLU 116, PRO 100, GLN 102 | Alkyl and Pi-Alkyl bond: CYS 101 |  | Pi – Sulfur: CYS 69, |
| 31 |  | Gamma-Tocopherol | -8.1 | - | Alkyl bond: ARG 103 | - | - |
| 32 |  | Genistein | -8.8 | GLN 102, GLU116 |  | Pi-anion bond: GLU116 |  |
| 33 |  | Genistin | -10.7 | GLN102, SER 99, GLU 116, | Alkyl bond: ARG 103 | Pi-anion bond: GLU 104 |  |
| 34 |  | Glyceryl ferulate | -6.6 | GLN 102, GLU 116, CYS 101, SER 99, PYR 115, GLU 116 | Alkyl bond: LYS 98 | Pi-anion bond: GLU 116 |  |
| 35 |  | Kaempferol | -9.1 | GLN 102, GLU 116, SER 99 |  | Pi- Anion bond: GLU 116 |  |
| 36 |  | p-Coumaric acid | -5.8 | PYR 115, SER 99, GLN 102 |  |  |  |
| 37 | AKT1 | 9,12-Octadecadienoic acid | -6.3 | THR 195 | Alkyl bond: VAL 164, ALA 177, ALA 230, LYS 179, LEU 181, LEU 156, MET 281, PHE 161, TYR 229, PHE 438 |  |  |
| 38 |  | Alatanin | -10.5 | GLU 228, ASN 279, ASP 292, GLY 311, GLY 157, GLU 278, ALA 230 | Pi-sigma bond: Leu 295. PI-PI T shaped bond: THE 161, ALA 177, ALA 230, LEU 156, MET 281, MET 227, LYUS 179, TYR 229, PHE 438, VAL 164, ALA 177 | Pi anion bond: ASP 292, | Pi-sulfur bond: MET 281 |
| 39 |  | Alpha-Tocopherol | -8.6 | GLU 228 | Alkyl bond: VAL 164, LEU 181, PHE 161, VAL 164, ALA 177 |  | Pi-Sulfur bond: MET 281, |
| 40 |  | Bumetrizole | -8.7 | ASP 292 | Amide and Alkyl bond: LYS 158, PHE 161, VAL 164, PHE 161, LYS 179 | Pi anion and Pi cation bond: LYS 179, ASP 292 | - |
| 41 |  | Cianidanol | -8.1 | GLU 278, LEU 156, THR 291 | Pi-Alkyl bond: VAL 164, ALA 177, ALA 230. |  | Pi-sulfur: MET 281 |
| 42 |  | Cinnamyl cinnamate | -7.9 | LYS 179 | Pi-Sigma, Pi-Pi T- shaped and Pi alkyl bond: VAL 164, Phe 161, LEU 181, MET 281 |  |  |
| 43 |  | Cycloartane | -7.8 |  | Pi-Sigma Alkyl and Pi-Alkyl bond: PHE236, LEU277, TYR350 |  |  |
| 44 |  | Daidzein | -8 | GLY 294 | **Pi sigma and Pi alkyl bond:** LEU 295, HIS 194 | **Pi-Anion bond:** ASP 292, GLU 191 |  |
| 45 |  | Delphinidin 3-glucoside | -7.7 | THR160, GLU 278, GLY 294, ASP 292 | Pi-Pi T shaped bond: PHE 161 | Pi anion bond: ASP 292 |  |
| 46 |  | Diosgenin | -8.8 | THR 195, LYS 276, THR 312, GLU 198 | Pi-alkyl bond: THE 161 |  |  |
| 47 |  | Epicatechin | -8.6 | ALA 230 | Pi sigma bond: MET 281 | Pi-Anion bond: GLU 234 | Pi sulfur bond: MET 281 |
| 48 |  | epidiosbulbin E acetate | -9.7 | GLY 162, THR 291 | Amide and Alkyl bond: GLY 159, THR 160, VAL 164, |  |  |
| 49 |  | gamma-Tocopherol | -7.9 |  | Alkyl bond: VAL 164, LYS 179, LEU 181, PHE 161, VAL 164, ALA 177 |  | Pi-Sulfur bond: MET 227, MET 281 |
| 50 |  | Genistein | -7.8 | THR195, GLU 198, GLY 294 | Pi-sigma and Pi-Alkyl bond: LEU 295, HIS 194 | Pi-anion bond: ASP 292, GLU 191 |  |
| 51 |  | Genistin | -8.9 | LYS 179, HIS 194, THR 291, GLY 311, GLY 162 | Pi sigma and Pi-Pi T shaped bond: LEU 295, PHE 161 | Pi-anion bond: ASP 292 |  |
| 52 |  | Glyceryl ferulate | -6.6 | LYS 179, ASP 292 | Alkyl and Pi-Alkyl bond: LEU 181, HIS 194, LEU 295, THE 161 | Pi-cation bond: LYS 179 |  |
| 53 |  | Kaempferol | -7.9 | LYS 179, GLY 294 | Pi-PI T-shaped and Pi-Alkyl bond: PHE 161, LEU 295, LYS 179, THE 161 | Pi-anion bond: GLU 181, ASP 274 |  |
| 54 |  | p-Coumaric acid | -6.3 | GLY 162, GLU 191 | Pi-PI T-shaped and Pi alkyl bond: THE 161, LEU 181 | Pi-cation bond: LYS 179 |  |
| 55 | PPARG | 9,12-Octadecadienoic acid | -5.7 | ARG288, SER 432 | Alkyl bond: PRO 227, ALA 292, ILE 336, LEU 330, LEU 228, MET 329, ARG 288 |  |  |
| 56 |  | Alatanin | -10.4 | GLN273, CYS 285, MET 348, GLU 272, GLY 284 | Pi-Sigma, Pi-alkyl and Alkyl bond: ARG288, ILE 326, LEU 330, LYS 275, ARG 280 |  |  |
| 57 |  | Alpha-Tocopherol | -8.3 |  | Pi-Sigma, Pi-alkyl and Alkyl bond: LEU 330, ARG 288, ALA 292, PRO 227, CYS 285 |  |  |
| 58 |  | Bumetrizole | -8.5 |  | Pi-alkyl and Alkyl bond: ALA 294, ILE 326, MET 329, CYS 285, ARG 288, ILE 341, ALA 292, LEU 330 |  | Pi-Sulfur bond: CYS 285 |
| 59 |  | Cianidanol | -8.1 | LEU 228 | Pi-alkyl bond: ARG 288, PRO 227 |  |  |
| 60 |  | Cinnamyl cinnamate | -8.4 |  | Pi-Sigma, Pi-Pi stacked alkyl andPi- Alkyl bond: LEU 465, PHE 363, MET 364, PHE 282, LEU 453, CYS 285, MET 463 |  |  |
| 61 |  | Cycloartane | -9.9 |  | Alkyl bond: MET 348, ILE 341, ARG 288, CYC 285, ILE 281, ALA 292, MET 329 |  |  |
| 62 |  | Daidzein | -8.1 |  | Pi-Sigma, Pi-Pi t shaped and Pi-Alkyl bond: LEU 465, GLN 286, HIS 449, LEU 459, LEU 453, MET 463 |  |  |
| 63 |  | Delphinidin 3-glucoside | -7.6 | ILE 472, THR 447, ARG 443 | Pi-Pi t Shaped and Pi Alkyl bond: TYR 320, VAL 446 |  |  |
| 64 |  | Diosgenin | -9.3 |  | Pi-alkyl and Alkyl bond: HYS 449, CYs 285, HYS 323, ALA 229, PRO 227, LEU 288. |  |  |
| 65 |  | Epicatechin | -8 | ARG 288, GLU 295, LEU 228 | **Pi-Alkyl bond:** PRO 227 | Pi-anion bond: GLU 295 |  |
| 66 |  | Epidiosbulbin E acetate | -9.3 |  | **Pi-Sigma, Pi-alkyl and Alkyl bond:** ILE 341, LEU 333, ARG 288, CYS 285, VAL 339 |  |  |
| 67 |  | Gamma-Tocopherol | -8.1 |  | **Pi-alkyl and Alkyl bond:** LEU 330, PRO 227, ALA 292, ILE 326, CYC 285, ARG 288 | Pi-Anion bond: GLU 295. |  |
| 68 |  | Genistein | -8.2 | ARG288, SER 342 | **Pi-Sigma and Pi-alkyl bond:** CYC 285, VAL 339, ALA 292 |  |  |
| 69 |  | Genistin | -9.5 | PHE 282 | **Pi-alkyl bond:** LEU 330, CYS 285, ARG 288, ALA 292, LEU 333, |  | Pi-sulfur bond: MET 364 |
| 70 |  | Glyceryl ferulate | -6.2 | ARG288, CYS 285, GLN 273, ARG 280 | **Pi-alkyl bond:** ILE 281 |  |  |
| 71 |  | Kaempferol | -8.1 | ALA 292, LEU 333, MET 329, LEU 330, ARG 288 |  |  |  |
| 72 |  | p-Coumaric acid | -6 | MET 463, GLU 460, THR 461 | **Pi-alkyl bond:** LEU 453, LYS 457, LEU 465 |  |  |
